# Supplementary material for: Archaeal and eukaryotic MCM rings sequentially melt DNA for replication initiation
Source: Nat Commun. 2026 Mar 31;17:4681. doi: 10.1038/s41467-026-70961-8 (PMC13201583; doi:10.1038/s41467-026-70961-8)
Supplement: Supplementary file 1 — Supplementary Information [file 41467_2026_70961_MOESM1_ESM.pdf]

Archaeal and eukaryotic MCM rings sequentially melt DNA for replication initiation

Supplementary information

Sanaz Rasouli[1,†], Alexander Myasnikov[1,†], Eric J. Enemark[1,2,3*]

Supplementary Figures 1-20 (pages 1-20)
Supplementary Tables 1-8 (pages 21-28)

[1]Department of Structural Biology, St Jude Children's Research Hospital, 262 Danny Thomas Place, Mail Stop 311, Memphis, TN 38105, USA

[2]Department of Biochemistry and Molecular Biology, University of Arkansas for Medical Sciences, 4301 W. Markham St., slot 516, Little Rock, AR 72205, USA

[3]Winthrop P. Rockefeller Cancer Institute, University of Arkansas for Medical Sciences, Little Rock, AR, 72205, USA

[†]These authors contributed equally to this work.

*To whom correspondence should be addressed: ejenemark@uams.edu

Present Address:
Sanaz Rasouli
Phenomenex Inc
411 Madrid Ave
Torrance, CA 90501
USA

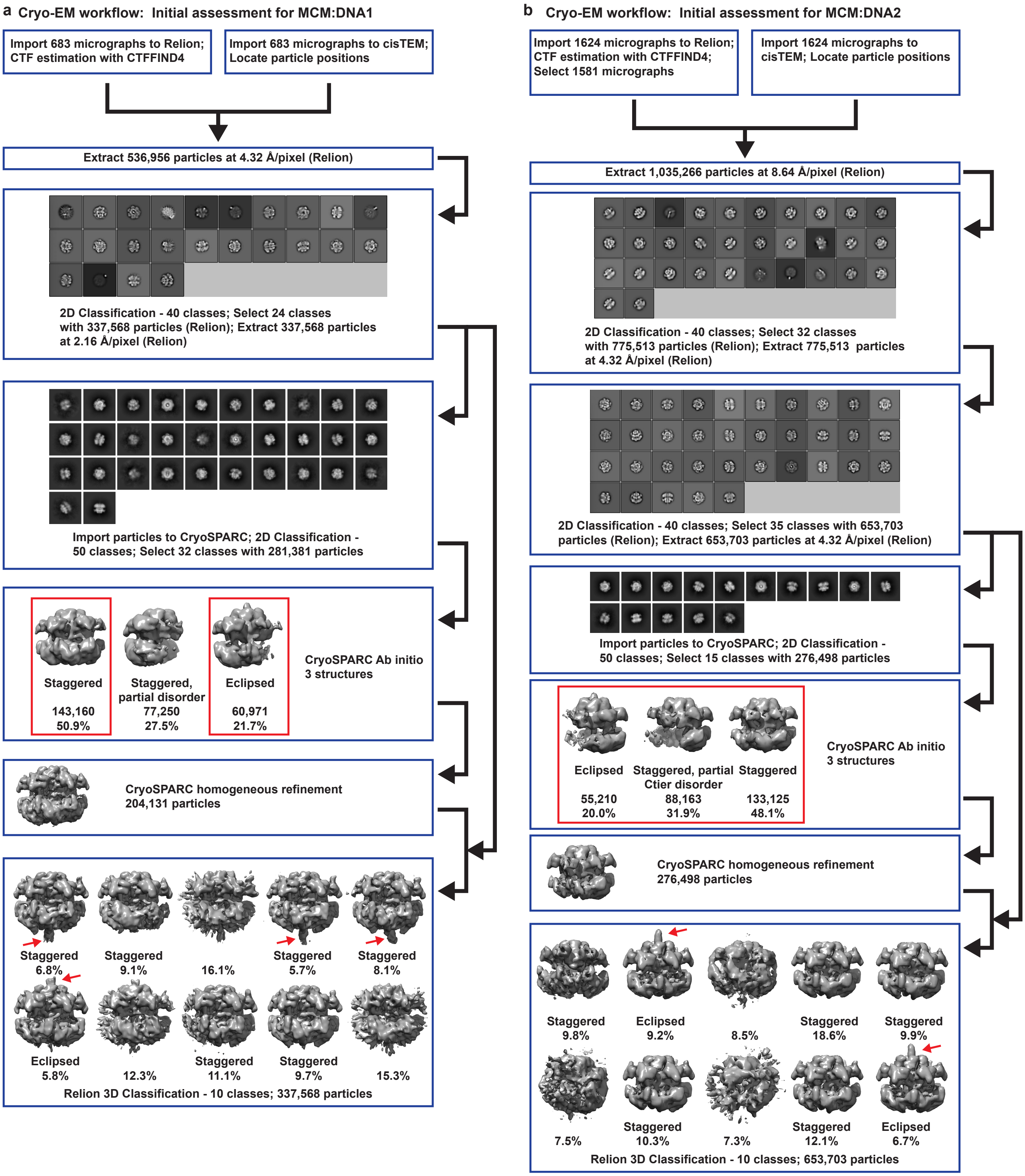

Supplementary Fig. 1: Initial assessment of MCM:DNA1 and MCM:DNA2.

a. Workflow for a subset of micrographs of the MCM:DNA1 dataset. **b.** Workflow for an initial MCM:DNA2 dataset. Both datasets identified staggered and eclipsed conformations. DNA was clearly present (red arrows) for a subset of 3D classes for both samples. Software used: Relion[125], CTFFind[126], cisTEM[127], Cryosparc[128], pyem[129], and Chimera[130].

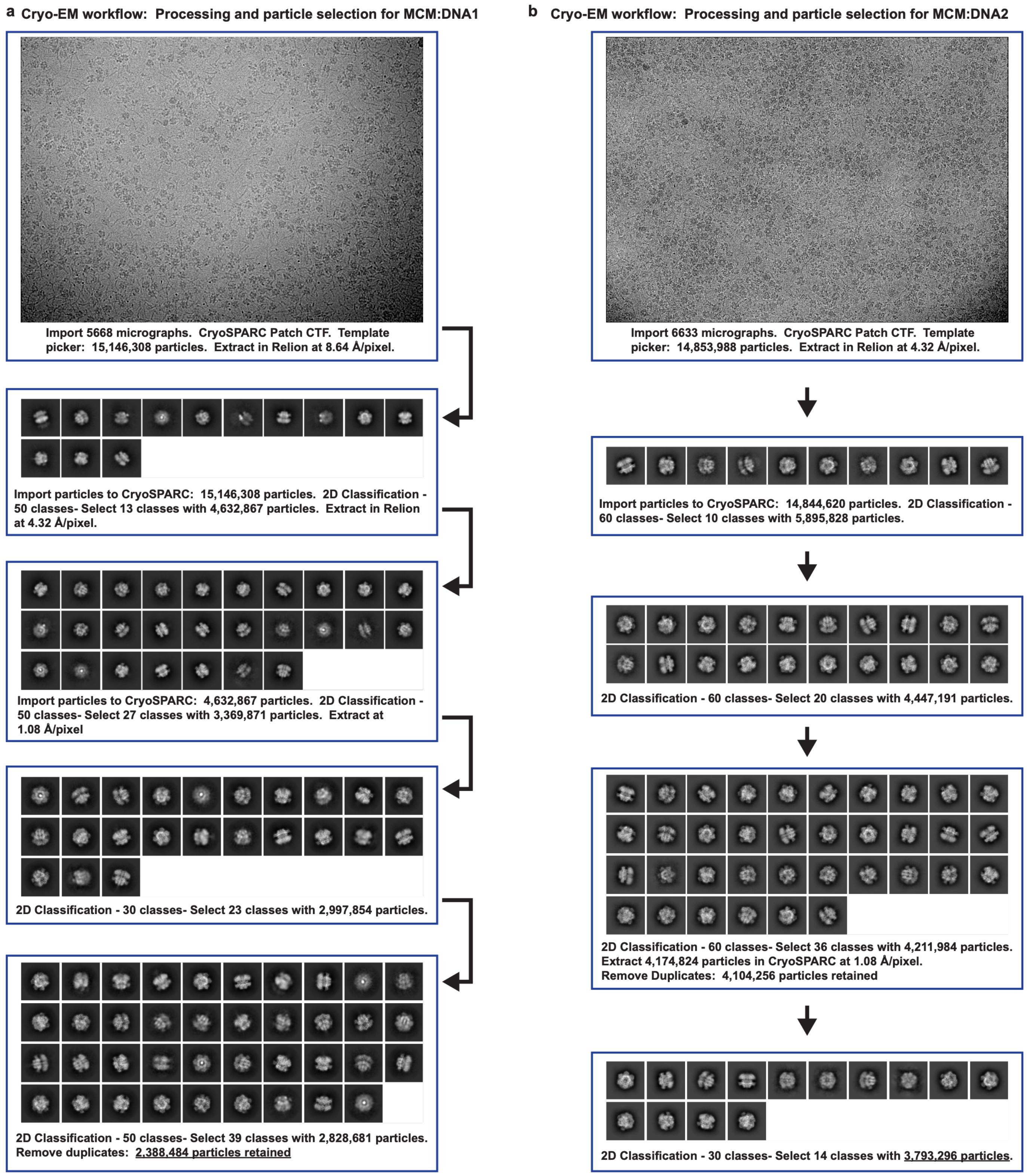

Supplementary Fig. 2: Processing and particle identification for MCM:DNA1 and MCM:DNA2.

a. Workflow for the full set of MCM:DNA1 micrographs. **b.** Workflow for the MCM:DNA2 sample. Both samples were subjected to four rounds of 2D classification to generate starting particle sets for 3D classification and refinement (see Supplementary Fig. 3-7). Software used: Cryosparc[128], pyem[129], Relion[125], and Chimera[130].

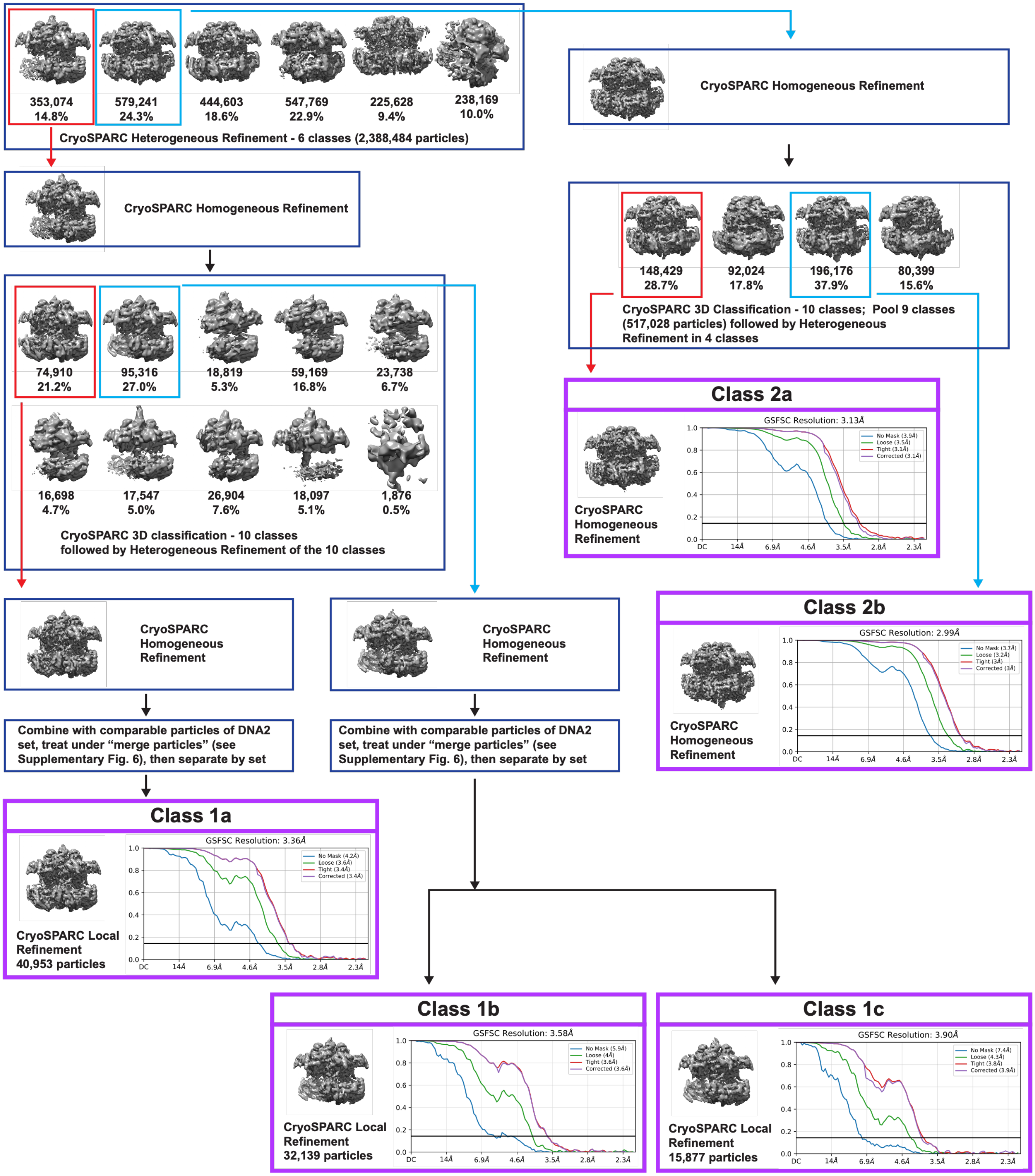

Supplementary Fig. 3: 3D Classification and refinement for MCM:DNA1 classes 1 and 2.

Initial 3D classification (top left) yielded an eclipsed conformation with internal DNA (red box, Class 1) and a staggered conformation with internal DNA (blue box, Class 2). Each was further differentiated to multiple subclasses. Class 1 subclasses were differentiated together with MCM:DNA2 (see Supplementary Fig. 6). Software used: Cryosparc[128] and Chimera[130].

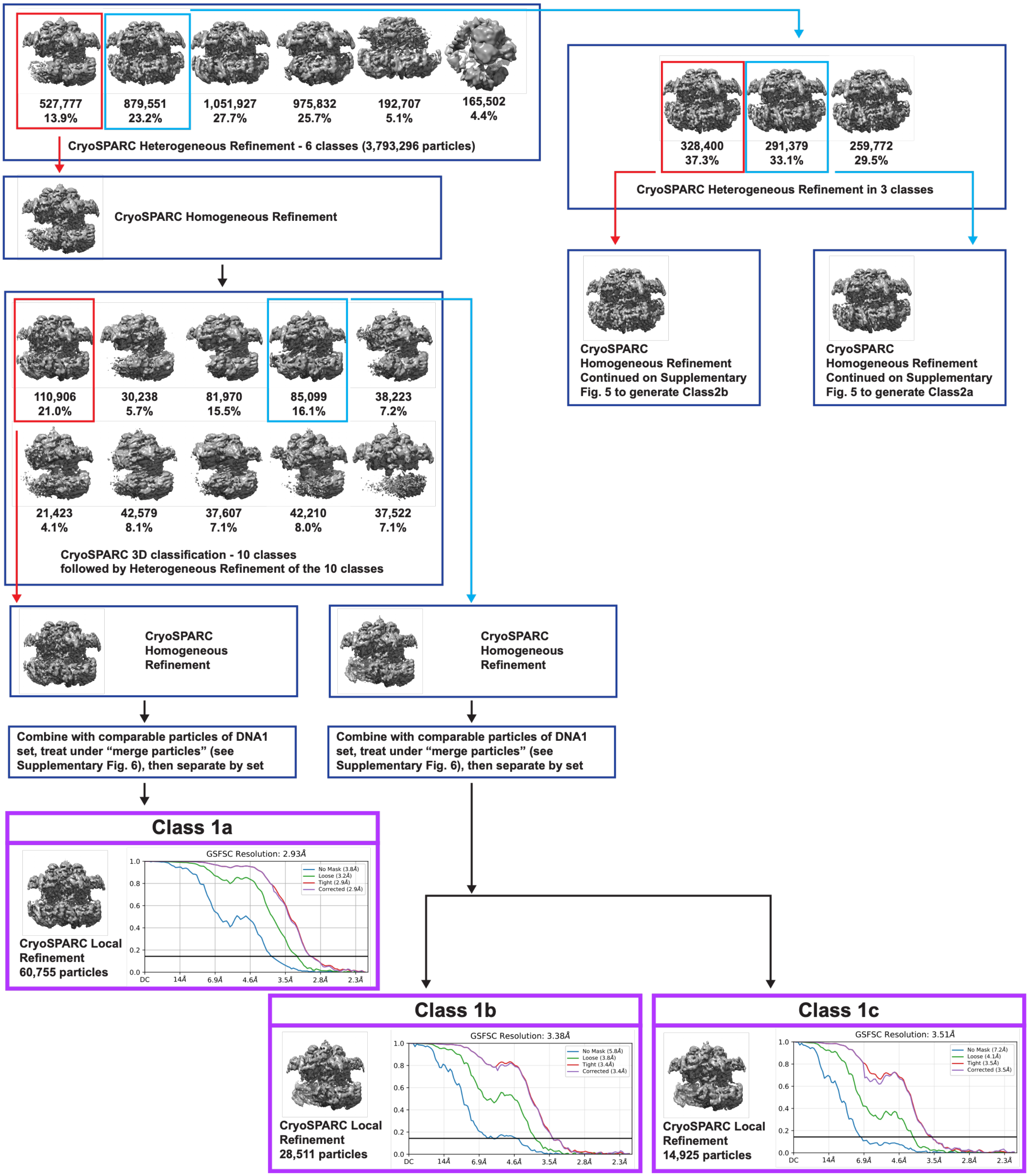

Supplementary Fig. 4: 3D Classification and refinement for MCM:DNA2 class 1 and 2 (part 1).

Initial 3D classification (top left) yielded an eclipsed conformation with internal DNA (red box, Class 1) and a staggered conformation with internal DNA (blue box, Class 2). Each was further differentiated to multiple subclasses. Class 1 subclasses were differentiated together with MCM:DNA1 (see Supplementary Fig. 6). Software used: Cryosparc[128] and Chimera[130].

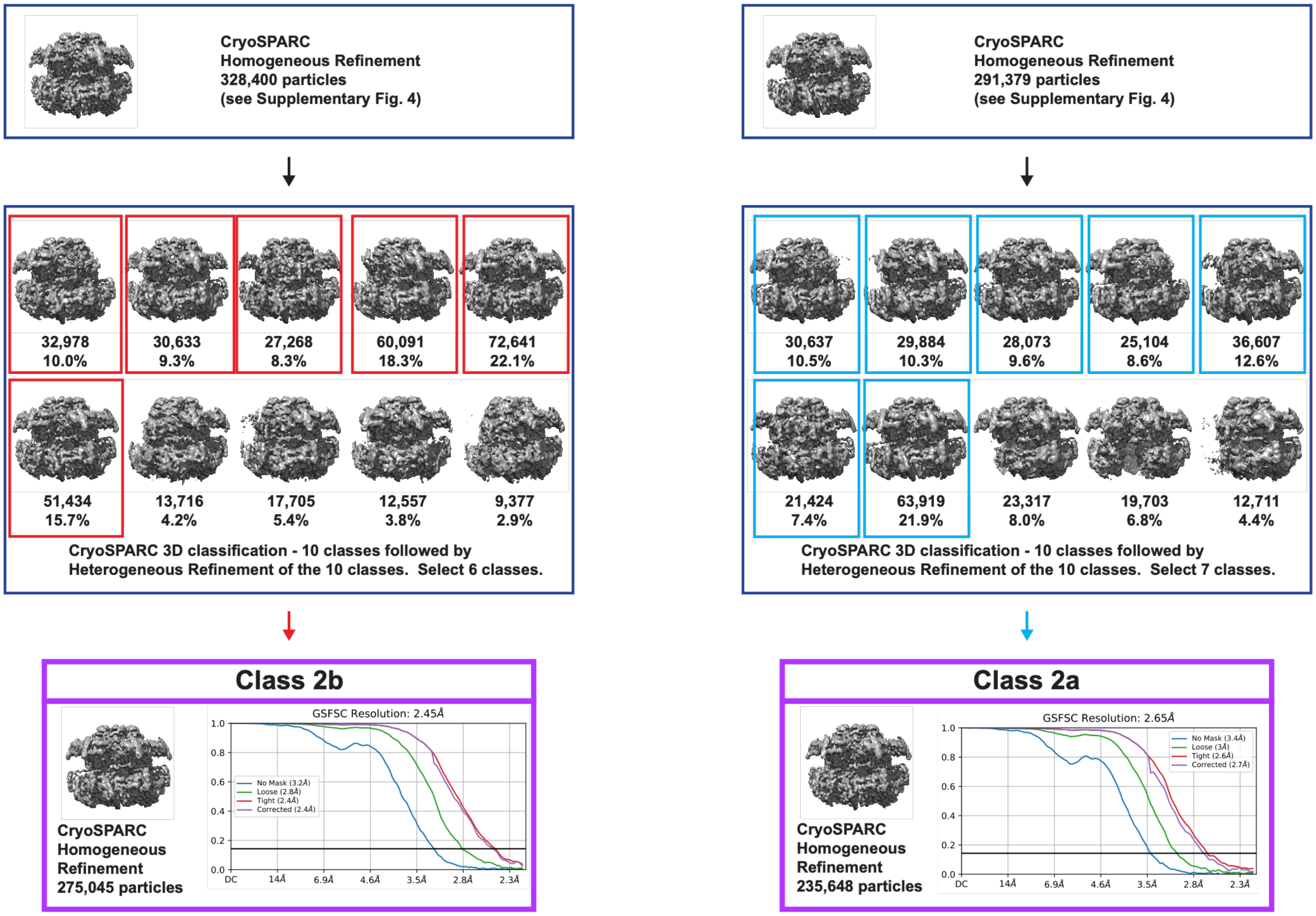

Supplementary Fig. 5: 3D Classification and refinement for MCM:DNA2 Class 2 (part 2).

Final particle sets for the MCM:DNA2 Class 2 structures were identified by 3D classification followed by heterogeneous refinement. Suitable classes were pooled for homogeneous refinement. Software used: Cryosparc[128] and Chimera[130].

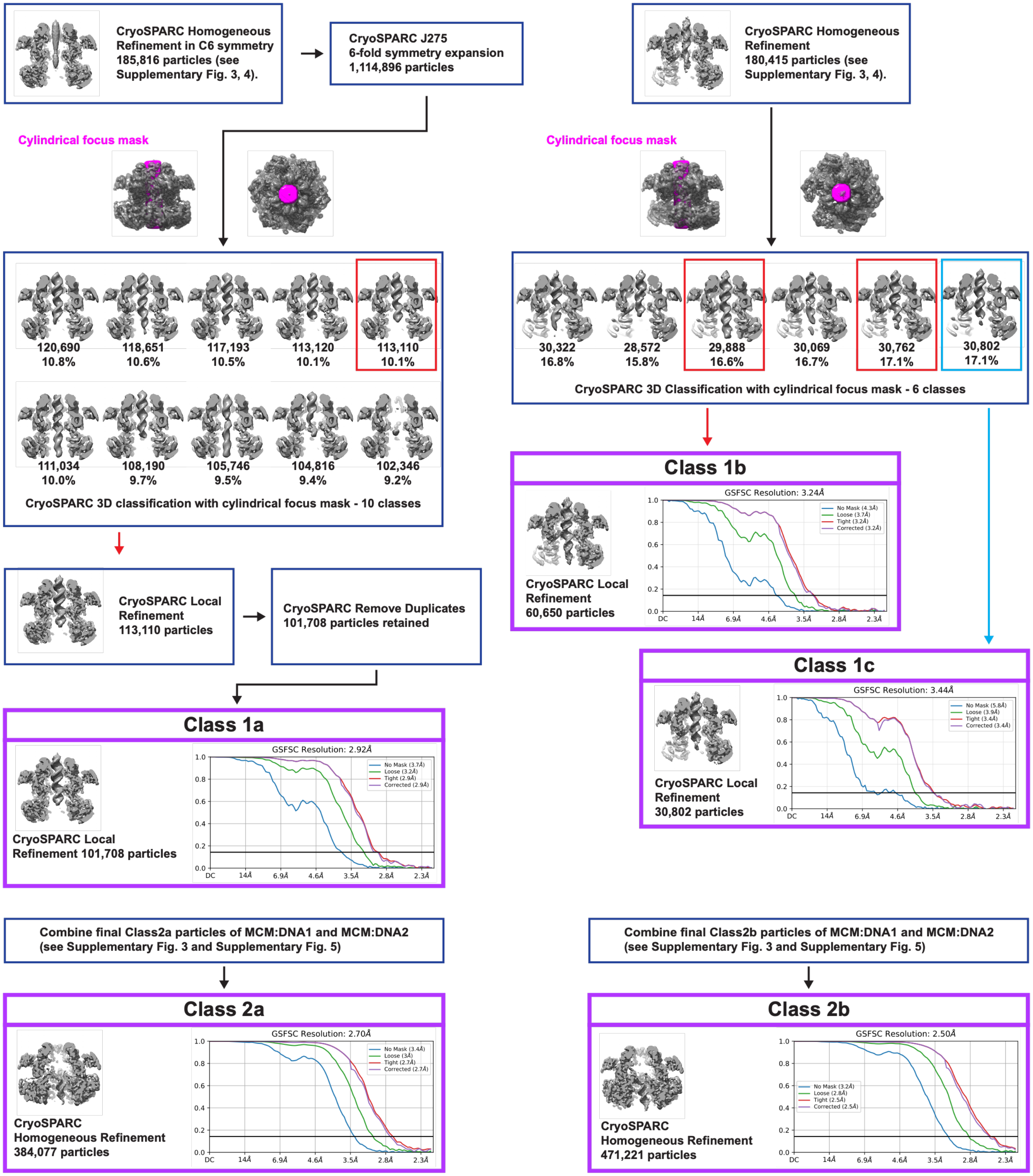

Supplementary Fig. 6: 3D Classification and refinement for merged particles classes 1 and 2.

Class 1 with 6 well-ordered ATPase domains (left) was refined in C6-symmetry. Particles were symmetry expanded about the C6-axis. Class 1 with 5 well-ordered ATPase domains (right) was refined in C1. Both were subjected to 3D classification with cylindrical focus mask (magenta) followed by local refinement of selected particles. Software used: Cryosparc[128] and Chimera[130].

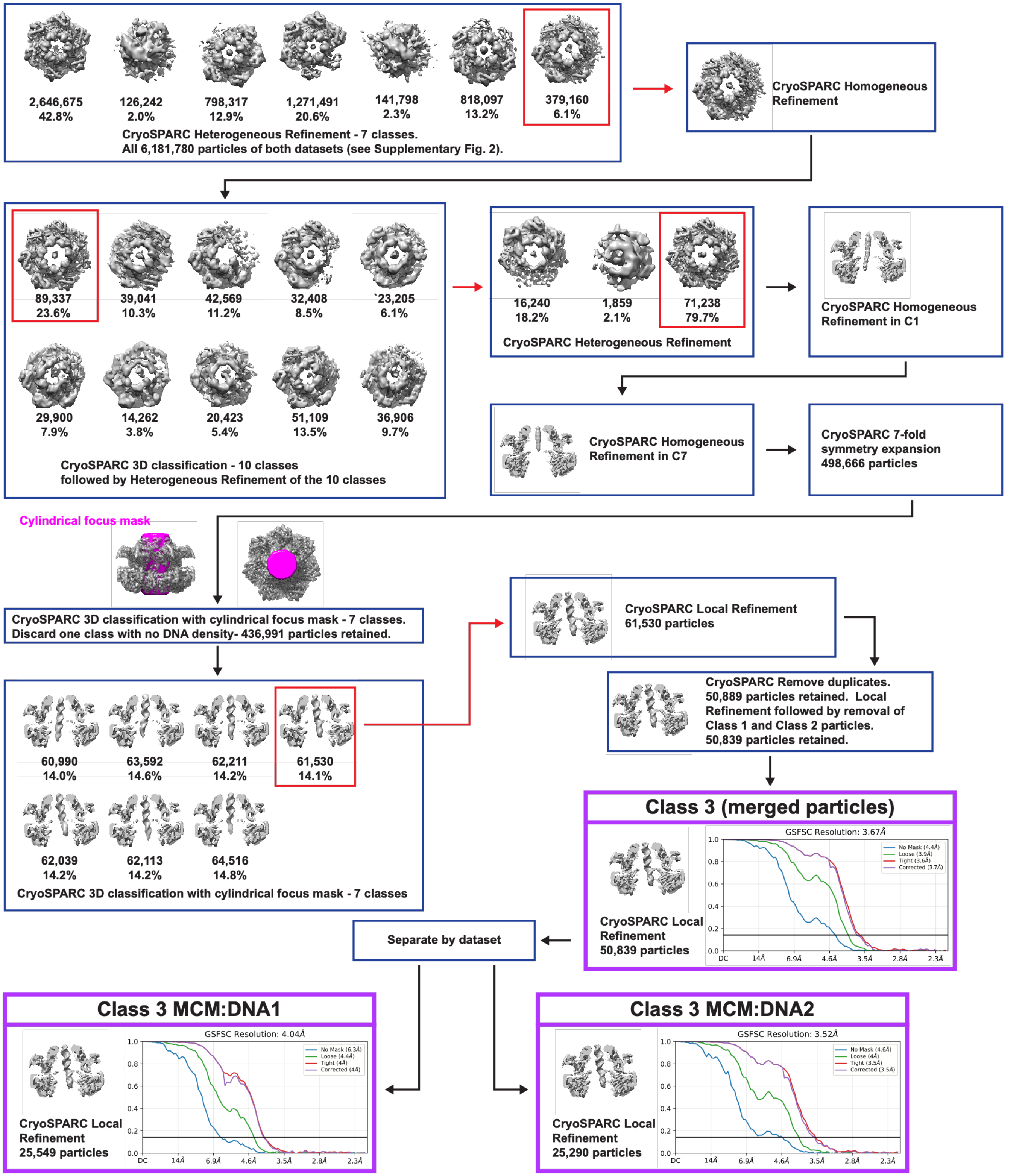

Supplementary Fig. 7: 3D Classification and refinement for all class 3 heptameric structures.

Class 3 heptameric particles were obtained by iterative 3D classification and refinement. The heptameric class was refined in C7-symmetry and symmetry expanded about the C7-axis. The structure was subjected to 3D classification with cylindrical focus mask (magenta) followed by local refinement. Software used: Cryosparc[128], Relion[125], pyem[129], and Chimera[130].

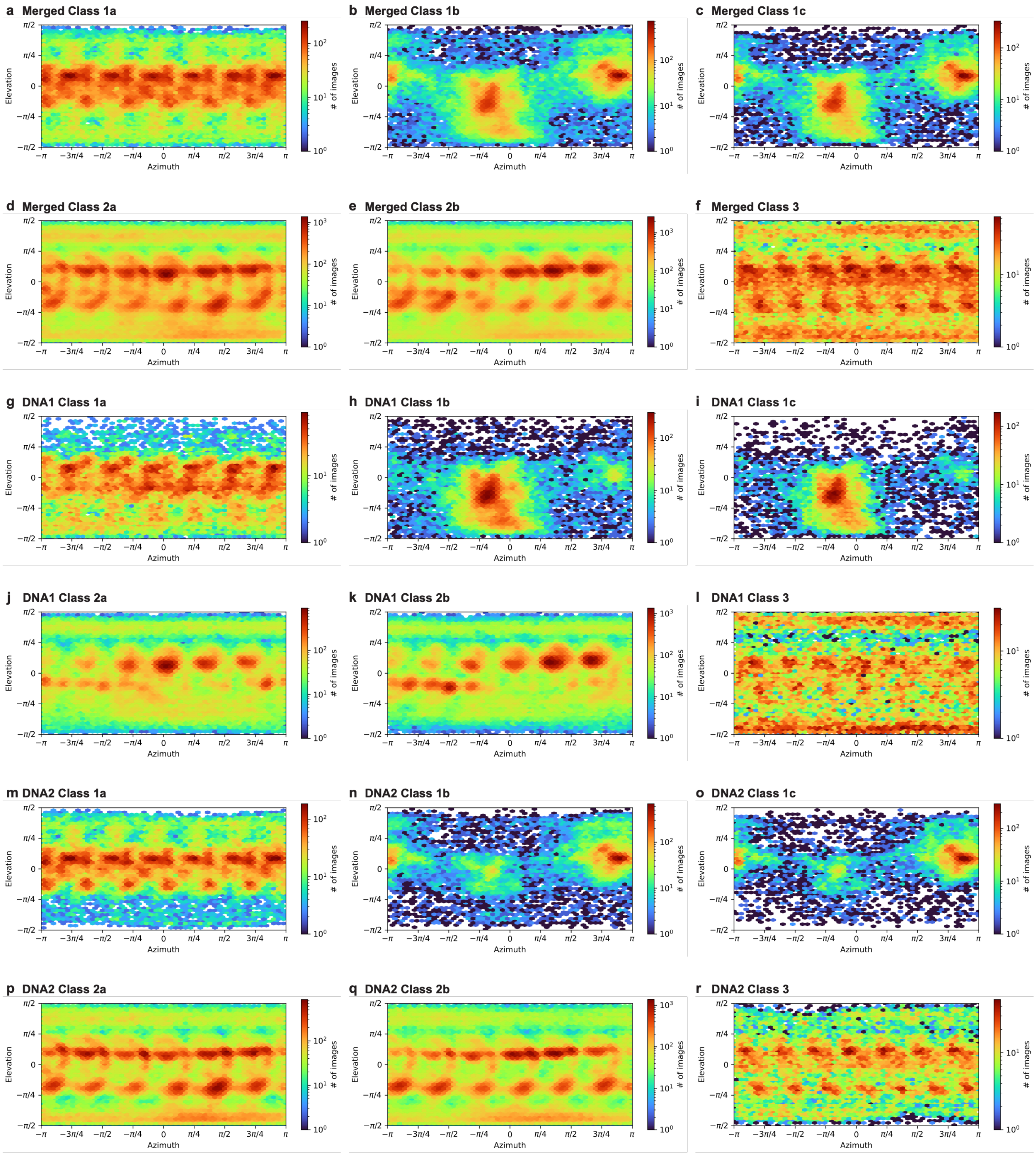

Supplementary Fig. 8: Angular distribution of particles used in the final reconstructions.

Angular distribution of particles for the Merged particle structures (**a-f**), structures with DNA1 (**g-l**), and structures with DNA2 (**m-r**) were determined and plotted by Cryosparc[128].

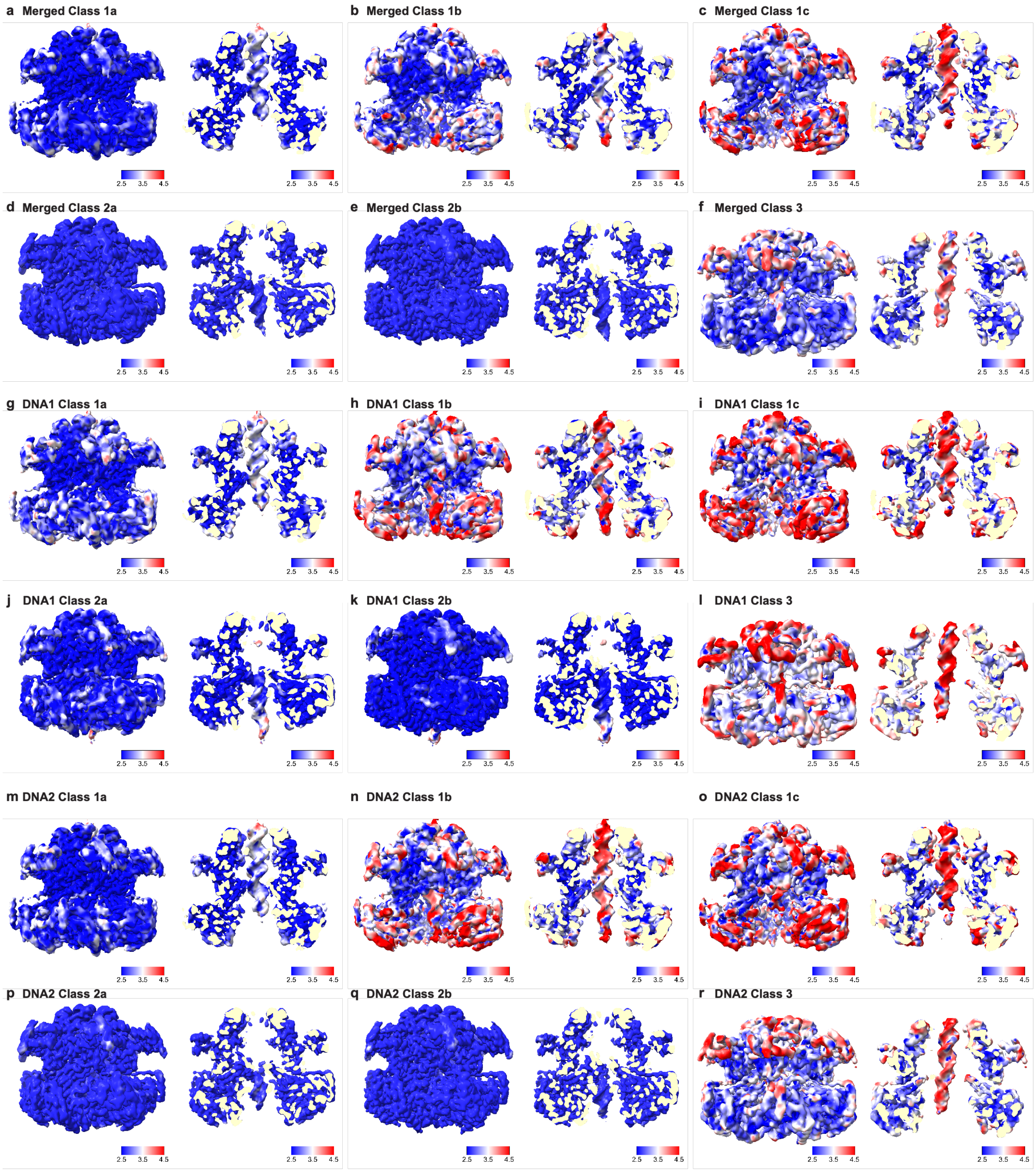

Supplementary Fig. 9: Local resolution maps for each structure.

Local resolution maps for the merged particle structures (**a-f**), structures with DNA1 (**g-l**), and structures with DNA2 (**m-r**) were calculated in Cryosparc[128] and mapped to the unsharpened maps and rendered in ChimeraX[136]. Each map is shown as unclipped (left) and clipped at front and back to highlight the DNA (right). Clipped surfaces are in light yellow.

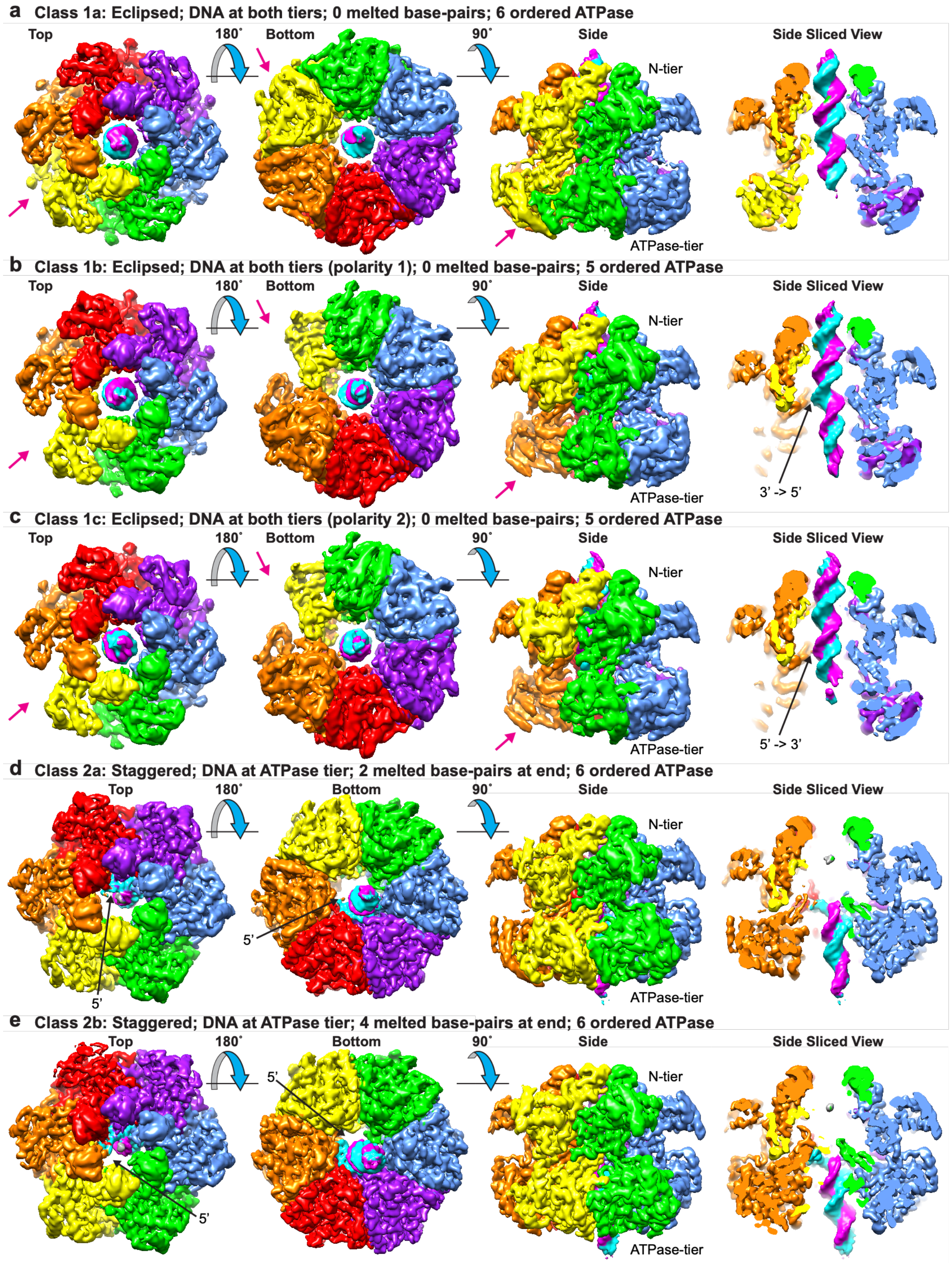

Supplementary Fig. 10: Overall architectures of the MCM hexamer:DNA1 complexes.

Major class 1 eclipses the two tiers and encircles duplex DNA at both tiers. Major class 2 staggers the two tiers and encircles partially melted DNA at the ATPase tier. **a.** Class1a: all 6 ATPase domains are well ordered, including the yellow subunit (magenta arrow). **b.** Class 1b: the ATPase domain of the yellow subunit (magenta arrow) is poorly ordered. The 3'->5' DNA strand (cyan) is near the orange hairpin (right panel). **c.** Class 1c: the ATPase domain of the yellow subunit (magenta arrow) is poorly ordered. The 5'->3' DNA strand (magenta) is near the orange hairpin (right panel). **d.** Class 2a: two terminal base-pairs are melted with the 5'-end of the cyan DNA strand near the red hairpins. **e.** Class 2b: four terminal base-pairs are melted with the 5'-end of the cyan DNA strand near the orange hairpins. Unsharpened maps are illustrated to emphasize the general MCM:DNA regions.

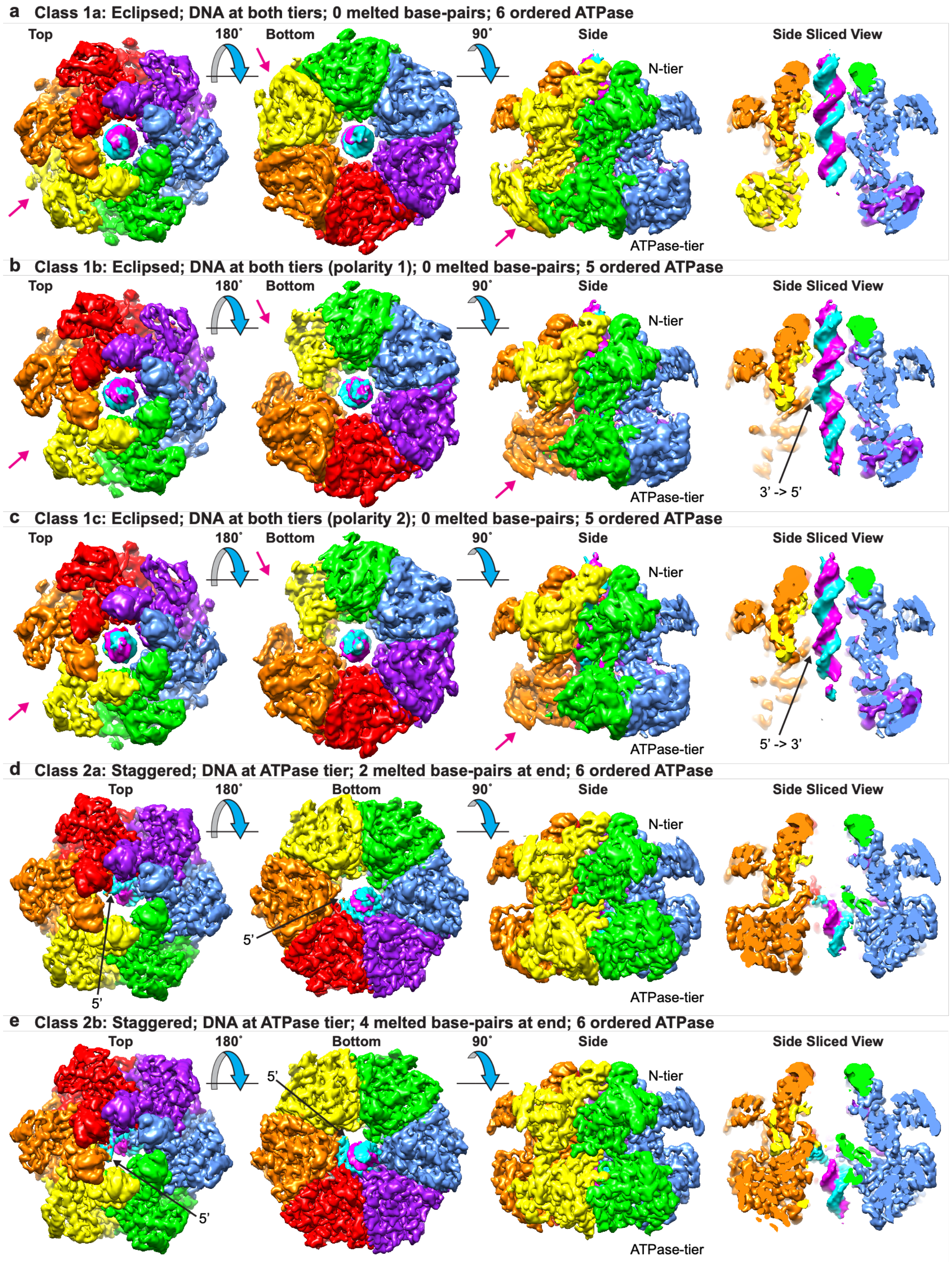

Supplementary Fig. 11: Overall architectures of the MCM hexamer:DNA2 complexes.

Major class 1 eclipses the two tiers and encircles duplex DNA at both tiers. Major class 2 staggers the two tiers and encircles partially melted DNA at the ATPase tier. **a.** Class1a: all 6 ATPase domains are well ordered, including the yellow subunit (magenta arrow). **b.** Class 1b: the ATPase domain of the yellow subunit (magenta arrow) is poorly ordered. The 3'->5' DNA strand (cyan) is near the orange hairpin (right panel). **c.** Class 1c: the ATPase domain of the yellow subunit (magenta arrow) is poorly ordered. The 5'->3' DNA strand (magenta) is near the orange hairpin (right panel). **d.** Class 2a: two terminal base-pairs are melted with the 5'-end of the cyan DNA strand near the red hairpins. **e.** Class 2b: four terminal base-pairs are melted with the 5'-end of the cyan DNA strand near the orange hairpins. Unsharpened maps are illustrated to emphasize the general MCM:DNA regions.

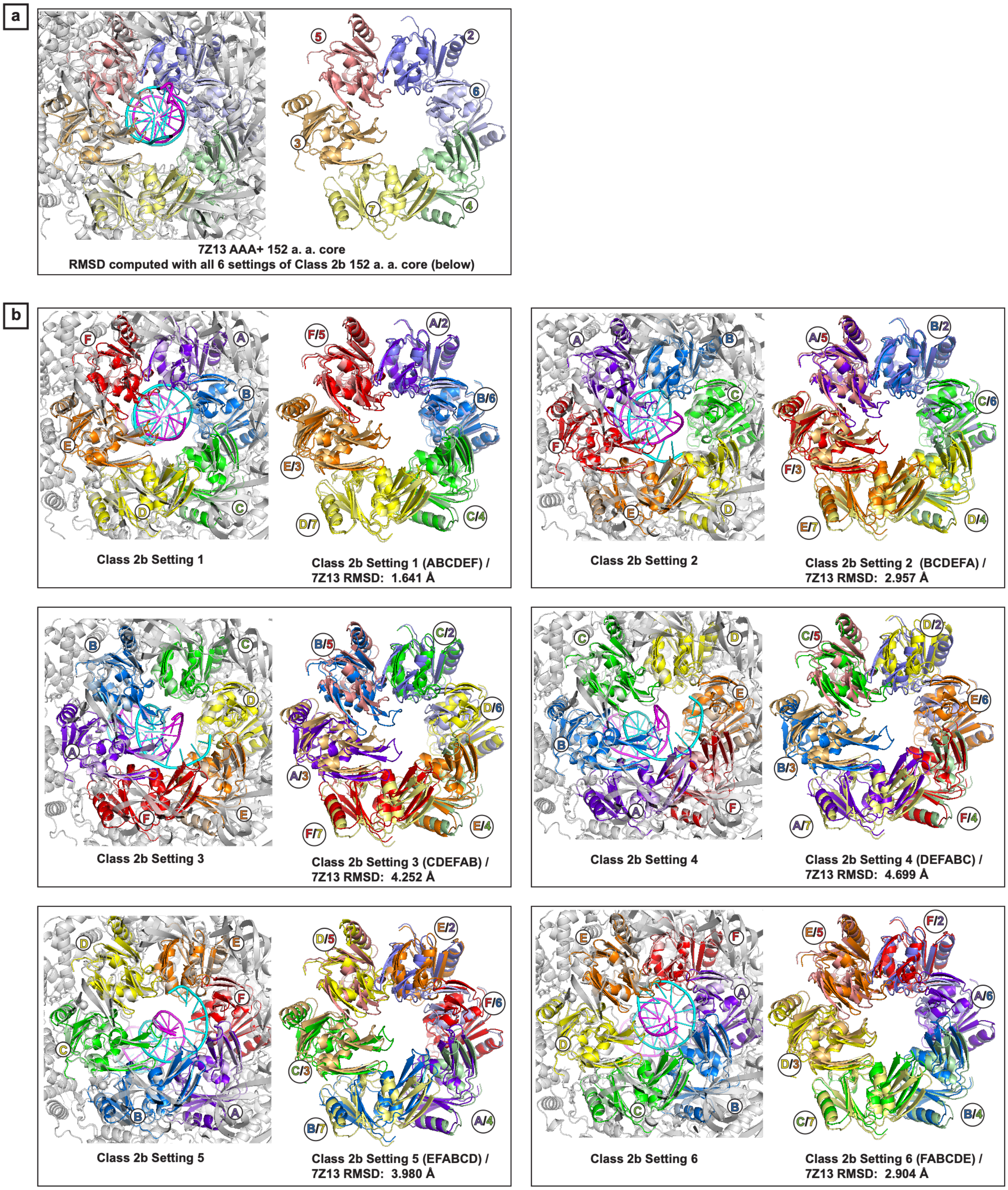

Supplementary Fig. 12: Six structure settings are possible when comparing the collective MCM ATPase tier architecture.

A highly conserved 152-amino acid core[16] provides the basis for comparing collective ATPase tier architectures. **a.** The hexameric core model of a comparative structure (such as PDB 7Z13[71]) is superimposed on that of each of permutation (Settings 1-6, panel **b**) of a reference model, such as Class 2b, and the RMSD is calculated in PyMOL[86]. Setting 1 (lowest RMSD) was selected for Fig. 6.

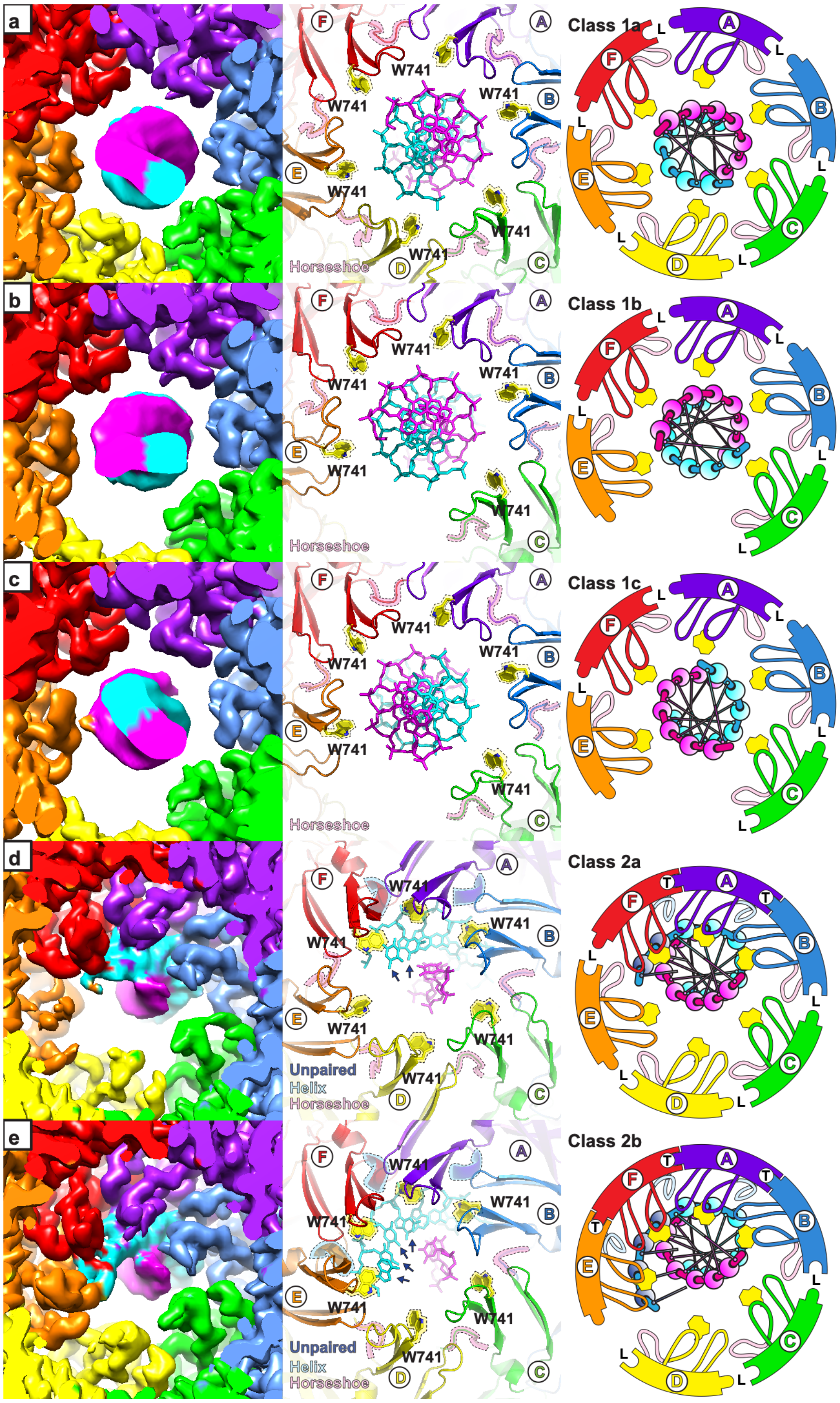

Supplementary Fig. 13: Comparison DNA-binding at the ATPase tier for MCM:DNA1.

Unsharpened map images are cutaway at a depth that emphasizes the ATPase DNA-binding hairpins. Aromatic wedge residues of the h2i are outlined and shaded yellow. For the Class 1 structures (**a-c**), the hairpins do not intimately contact either DNA strand, and each h2i hairpin adopts a horseshoe conformation (outlined and shaded pink in central panels) to create a loose intersubunit interface (L in right panel cartoon). For the Class 2 structures (**d-e**), the subunits where the h2i hairpins adopt a helix conformation (outlined and shaded blue in central panels) intimately associate with the cyan DNA strand and have a tight intersubunit interface (T in right panel cartoon). Two base-pairs are melted in Class 2a, and four are melted in Class 2b. Model chain identifiers (A-F) are provided in the middle and right panels.

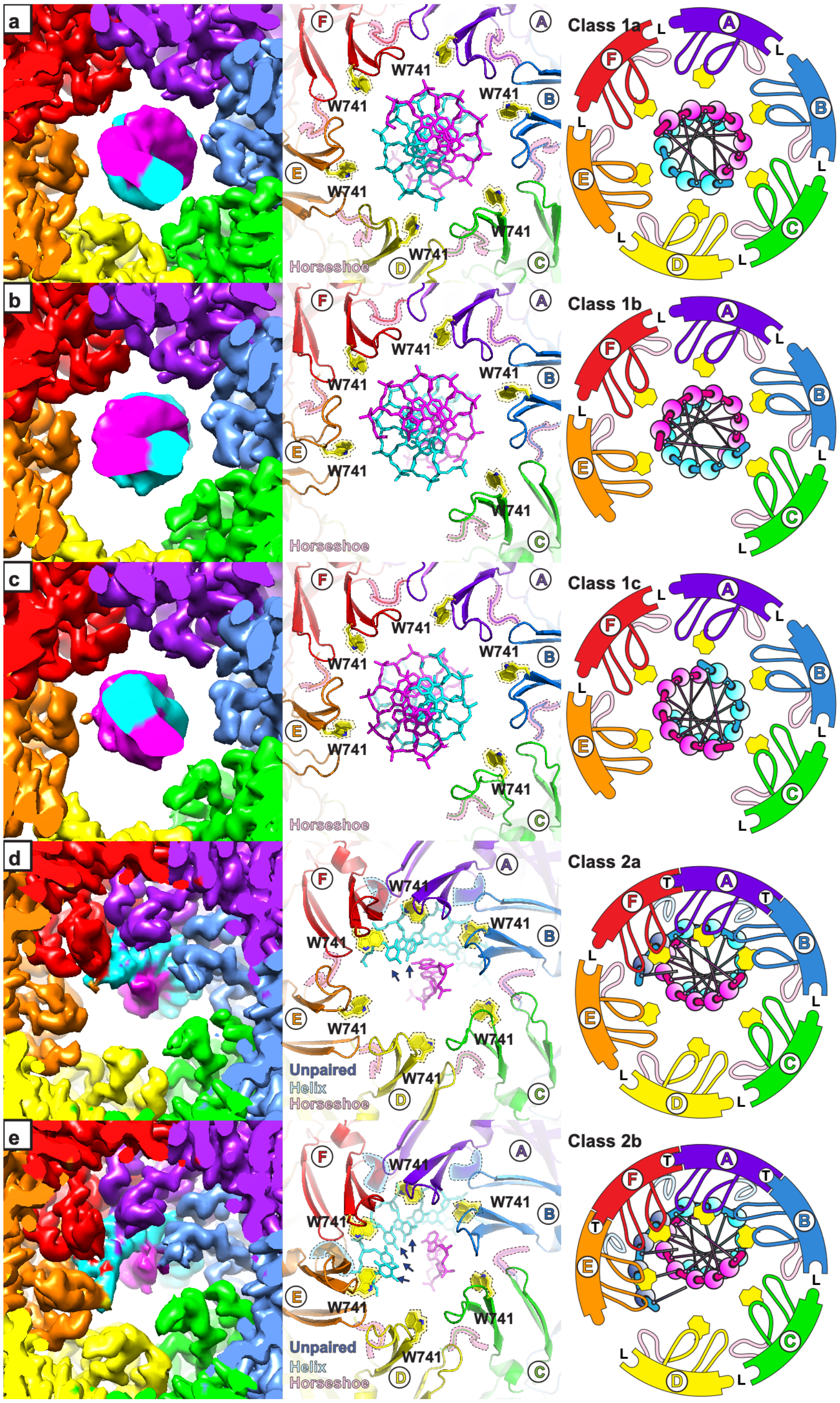

Supplementary Fig. 14: Comparison DNA-binding at the ATPase tier for MCM:DNA2.

Unsharpened map images are cutaway at a depth that emphasizes the ATPase DNA-binding hairpins. Aromatic wedge residues of the h2i are outlined and shaded yellow. For the Class 1 structures (**a-c**), the hairpins do not intimately contact either DNA strand, and each h2i hairpin adopts a horseshoe conformation (outlined and shaded pink in central panels) to create a loose intersubunit interface (L in right panel cartoon). For the Class 2 structures (**d-e**), the subunits where the h2i hairpins adopt a helix conformation (outlined and shaded blue in central panels) intimately associate with the cyan DNA strand and have a tight intersubunit interface (T in right panel cartoon). Two base-pairs are melted in Class 2a, and four are melted in Class 2b. Model chain identifiers (A-F) are provided in the middle and right panels.

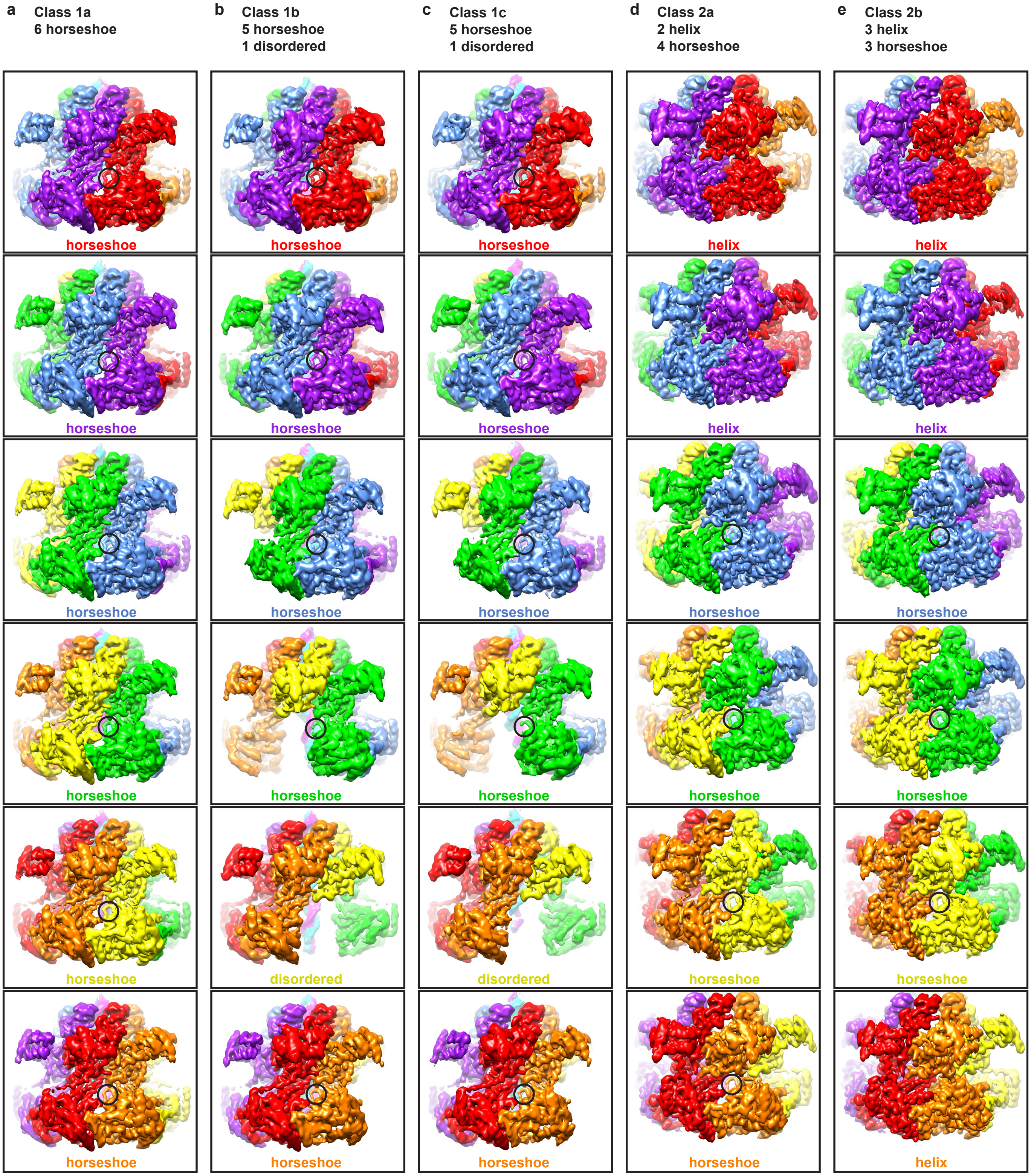

Supplementary Fig. 15: The horseshoe conformation is readily identified in side-views.

Class 1 structures (**a-c**) have horseshoe conformations at each ordered subunit. Class 2 structures (**d-e**) include both helix and horseshoe conformations. For the horseshoe arrangement, the interface is more open, making the horseshoe directly visible from the side (black circle). The interface involving the helix conformation is much tighter such that the helix is occluded in the side view. Subunits are colored as in Fig. 1.

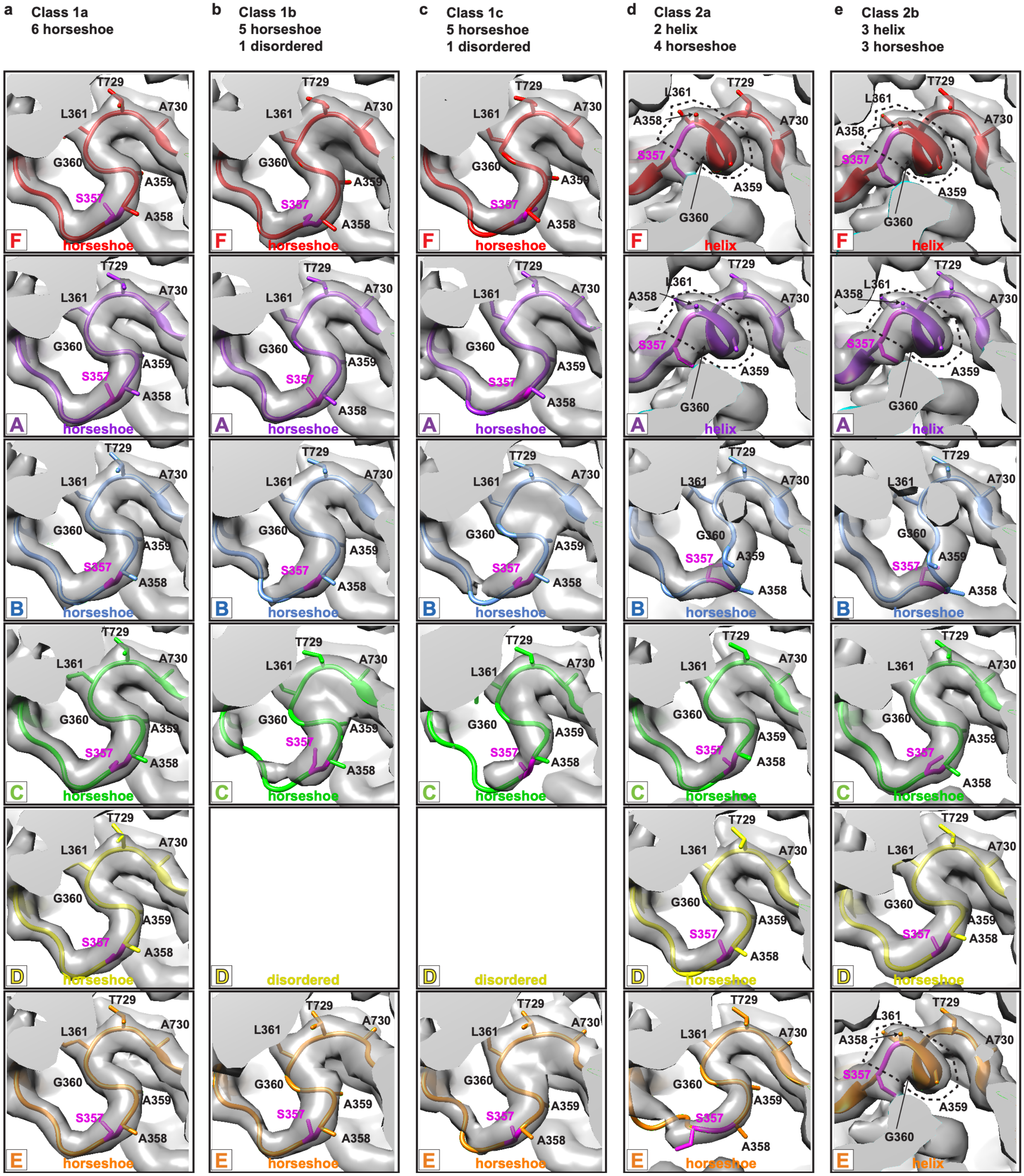

Supplementary Fig. 16: Horseshoe and helix conformations for the merged particle structures.

Images for structures (**a-e**) are with a consistent perspective of the helical portion of helix-2 (residues A745-A751, below plane of view) that directly follows the insert hairpin of the h2i. Residues that precede the insert hairpin (S357-L361/T729) adopt horseshoe or helix (dashed outline) conformations. S357 with a conserved hydroxyl that can interact with DNA is in magenta. Maps are drawn with 50% transparency. Chain identifiers (A-F) are provided for each panel. L361 is directly linked to T729 due to intein removal.

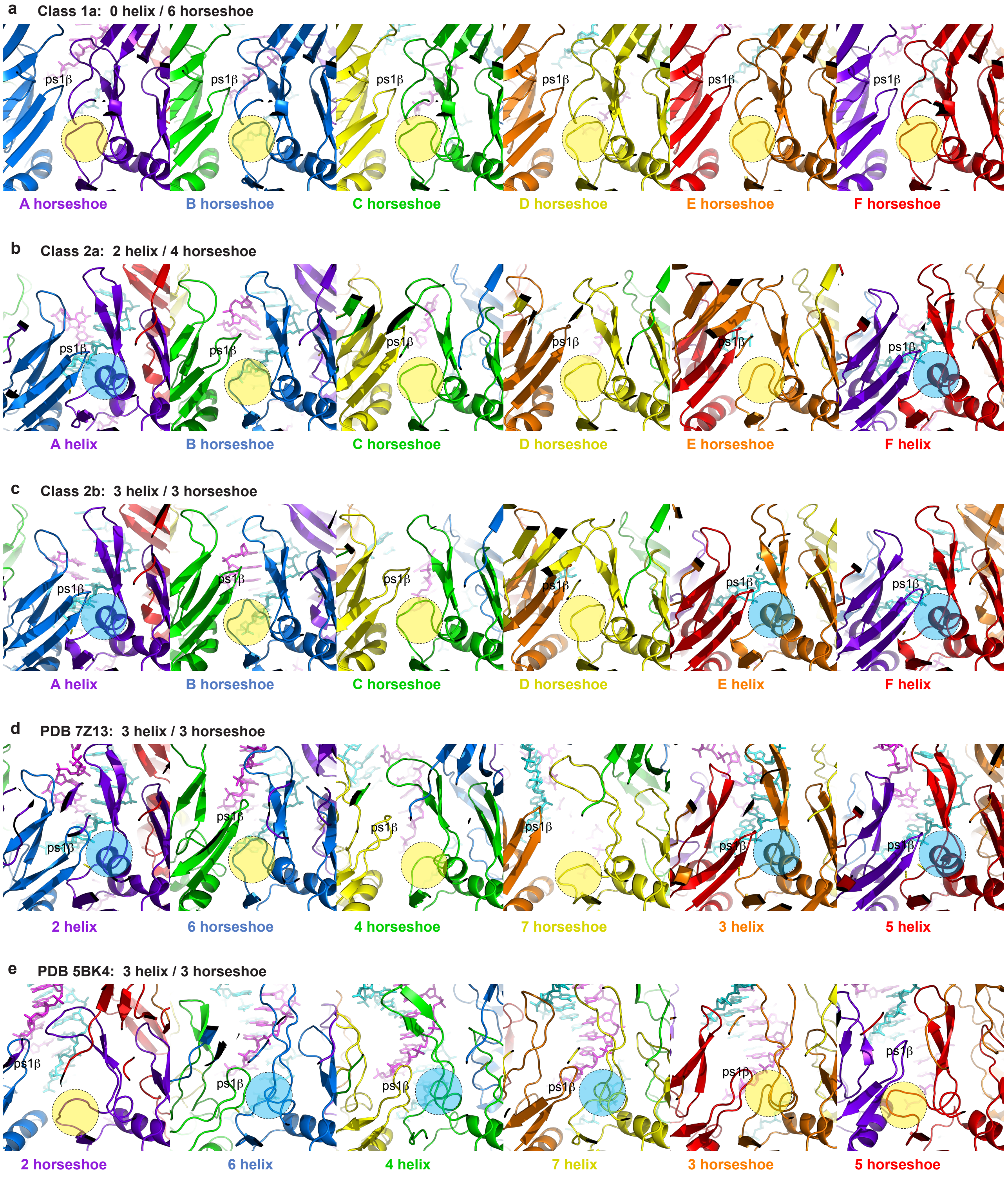

Supplementary Fig. 17: The helix/horseshoe conformations of each subunit interface.

Helix (blue circles) permits the h2i to get closer to the adjacent subunit ps1β than horseshoe (yellow circles). Class 1a (**a**) has 0 and Class 2a (**b**) has 2 helix conformations. The 3 consecutive helix conformations of Class 2b (**c**) matches the arrangement of *Sc*CMG bound to melted DNA (PDB 7Z13[71]) (**d**) and appears opposite *Sc*Mcm2-7 double-hexamer encircling intact dsDNA (PDB 5BK4[37]) (**e**). Chain identifiers (A-F or 2, 6, 4, 7, 3, 5) are below each panel.

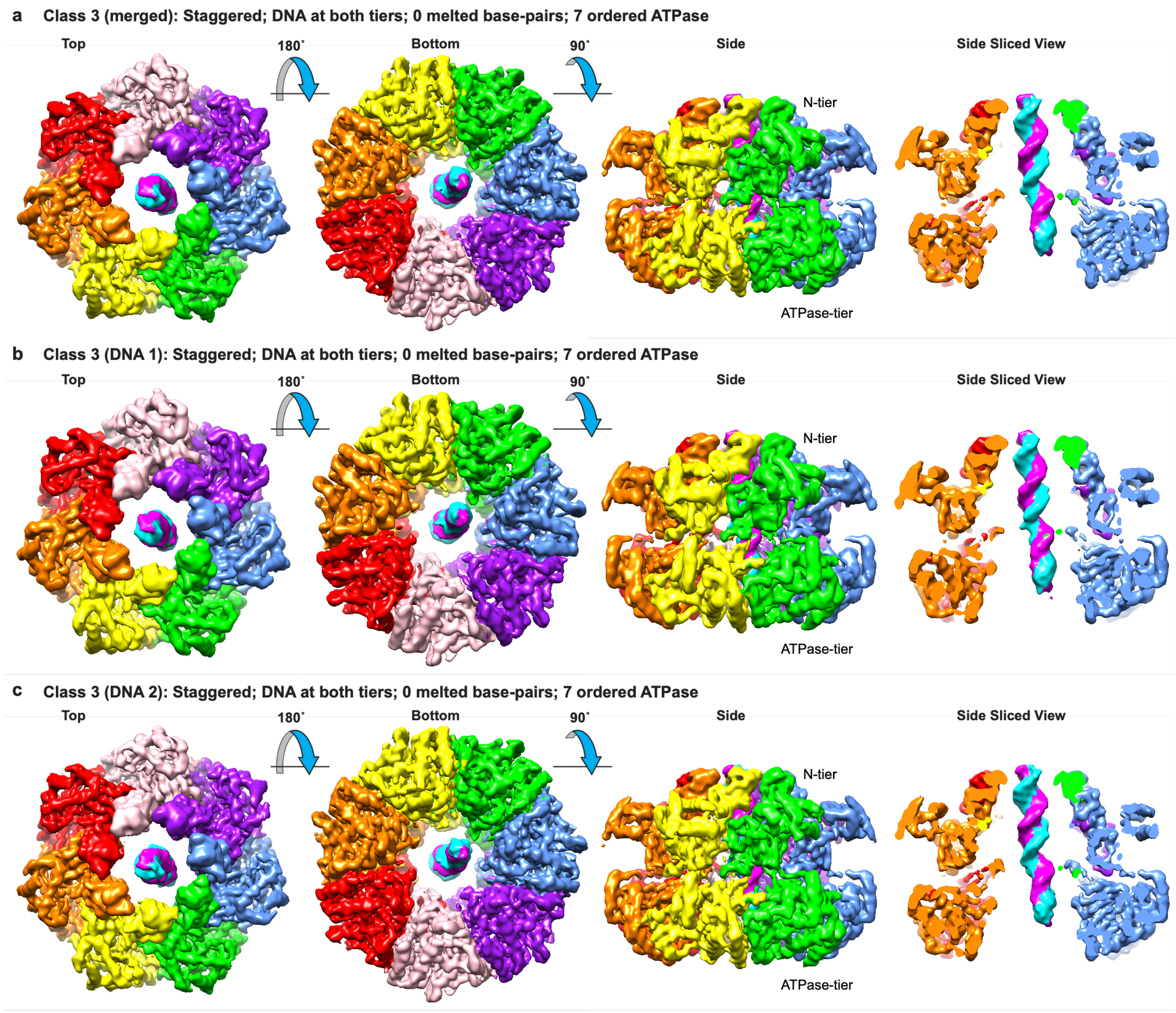

Supplementary Fig. 18: Overall architectures of the MCM heptamer:DNA complexes.

The two tiers are staggered and encircle duplex DNA at both tiers. **a.** The heptameric complex obtained from merging the particles of both DNA samples. **b**. The heptameric complex obtained from DNA1. **c**. The heptameric complex obtained from DNA2. Unsharpened maps are illustrated to emphasize the general MCM:DNA regions.

WP_009991364.1	*Sso*MCM	421	M E Q Q T V S I A K A G I V A K L N A R
AAL80606.1	*Pf*MCM	776	L E Q Q T I S I S K A G I T A T L N A R
WP_011277765.1	*Sa*MCM	421	M E Q Q T V S I A K A G I V A K L N A R
WP_048061327.1	*Mt*MCM	398	L E Q Q T I S I A K A G I M A T L N S R
WP_148679238.1	*Ap*MCM	418	M E Q Q T V S I A K A G I K A T L S A R
WP_011250571.1	*Tk*MCM	550	L E Q Q T I S I S K A G I T A T L N S R
P29496	*Sc*Mcm5	497	M E Q Q T I S I A K A G I T T V L N S R
P33992	*Hs*Mcm5	462	M E Q Q T I S I A K A G I T T T L N S R
P41389	*SpMcm5*	453	M E Q Q T I S I A K A G I T T I L N S R
Q9VGW6	*Dm*Mcm5	459	M E Q Q T I S I A K A G I T T T L N S R
Q21902	*Ce*Mcm5	461	M E Q Q T I S I A K A G I T T T L N S R
P55862	*Xl*Mcm5	463	M E Q Q T I S I A K A G I T T T L N S R
Q6NV07	*Dr*Mcm5	464	M E Q Q T I S I A K A G I T T T L N S R
P49718	*Mm*Mcm5	462	M E Q Q T I S I A K A G I T T T L N S R
O80786	*At*Mcm5	456	M E Q Q T I S I A K A G I T T V L N S R
Q54CP4	*Dd*Mcm5	450	M E Q Q T I S I A K A G I T T I L N S R
A0A8U0WNY5	*Eh*Mcm5	439	M E Q Q T I S I A K A G I T A V L N A R
EET02444.1	*Gd*Mcm5	466	M E Q G S I S I S K A G I S A T L N A R
P29469	*Sc*Mcm2	624	M E Q Q S I S I S K A G I V T T L Q A R
P49736	*Hs*Mcm2	604	M E Q Q S I S I S K A G I V T S L Q A R
P40377	*Sp*Mcm2	615	M E Q Q S I S I S K A G I V T T L Q A R
P49735	*Dm*Mcm2	589	M E Q Q S I S I S K A G I V T S L Q A R
Q9XXI9	*Ce*Mcm2	579	M E Q Q S I S I S K A G I V T S L H A R
P55861	*Xl*Mcm2	589	M E Q Q S I S I S K A G I V T S L Q A R
A0A0R4IF65	*Dr*Mcm2	592	M E Q Q S I S I S K A G I V T S L Q A R
P97310	*Mm*Mcm2	604	M E Q Q S I S I S K A G I V T S L Q A R
Q9LPD9	*At*Mcm2	630	M E Q Q S I S I S K A G I V T S L Q A R
Q54LI2	*Dd*Mcm2	714	M E Q Q S I S I S K A G I V T T L T A R
C4LYY1	*Eh*Mcm2	669	M E Q Q S I S I S K A G I V T S L K A R
EES98420.1	*Gd*Mcm2	301	M E Q Q T V S V A K A G I I S T L E A R
P53091	*Sc*Mcm6	656	M E Q Q T I S I A K A G I H A T L N A R
Q14566	*Hs*Mcm6	477	M E Q Q T I S I T K A G V K A T L N A R
P49731	*Sp*Mcm6	557	M E Q Q T I S I A K A G I Q A T L N A R
Q9V461	*Dm*Mcm6	469	M E Q Q T I S I A R A G V R A T L N A R
P34647	*Ce*Mcm6	478	M E Q Q T I S I T K A G V K A T L N A R
Q5FWY4	*Xl*Mcm6	479	M E Q Q T I S I T K A G V K A T L N A R
Q4V9J1	*Dr*Mcm6	471	M E Q Q T I S I T K A G V K A T L N A R
P97311	*Mm*Mcm6	477	M E Q Q T I S I T K A G V K A T L N A R
F4KAB8	*At*Mcm6	476	M E Q Q T I S I T K A G I Q A T L N A R
Q86B14	*Dd*Mcm6	551	M E Q Q T I S I A K A G I H A S L N A R
C4LYN5	*Eh*Mcm6	475	M E Q Q T I S I A K G G L H A T L N A R
EET01045.1	*Gd*Mcm6	565	L E Q Q S V S I N K A G I S I T L K A K
P30665	*Sc*Mcm4	649	M E Q Q T I S I A K A G I I T T L N A R
P33991	*Hs*Mcm4	591	M E Q Q T L S I A K A G I I C Q L N A R
P29458	*Sp*Mcm4	626	M E Q Q T V T V A K A G I I T T L N A R
Q26454	*Dm*Mcm4	593	M E Q Q T L S I A K A G I I C Q L N A R
Q95XQ8	*Ce*Mcm4	550	M E Q Q T L S I A K A G I I C Q L N A R
Q5XK83	*Xl*Mcm4	586	M E Q Q T L S I A K A G I I C Q L N A R
Q6NZV2	*Dr*Mcm4	573	M E Q Q T L S I A K A G I I C Q L N A R
P49717	*Mm*Mcm4	590	M E Q Q T L S I A K A G I I C Q L N A R
Q0WVF5	*At*Mcm4	566	M E Q Q T V S I A K A G I I A S L N A R
Q86IF1	*Dd*Mcm4	614	M E Q Q T V S I A K A G I I C T L N A R
C4M9H9	*Eh*Mcm4	397	M E Q Q T I S V A K S G I V C S L N A R
EET01291.1	*Gd*Mcm4	523	M E H G Q L S I A K A G I L A T L S A R
P38132	*Sc*Mcm7	541	M E Q Q T I S I S K A G I N T T L N A R
P33993	*Hs*Mcm7	462	M E Q Q T I S I A K A G I L T T L N A R
O75001	*Sp*Mcm7	484	M E Q Q T I S I S K A G I T T T L N A R
Q9XYU0	*Dm*Mcm7	462	M E Q Q T I S I A K A G I M T T L N A R
O16297	*Ce*Mcm7	471	M E Q Q T I S I A K A G I M T T L N A R
Q91876	*Xl*Mcm7	461	M E Q Q T I S I A K A G I M T T L N A R
Q7ZVL6	*Dr*Mcm7	462	M E Q Q T I S I A K A G I M T S L N A R
Q61881	*Mm*Mcm7	462	M E Q Q T I S I A K A G I L T T L N A R
P43299	*At*Mcm7	457	M E Q Q T V S I A K A G I T T S L N A R
Q54RU0	*Dd*Mcm7	534	M E Q Q T I S I A K A G I T T T L N A R
C4M2D0	*Eh*Mcm7	477	M E Q Q S I S I A K A G I T T S L N A R
EET00456.1	*Gd*Mcm7	445	M E Q G T I S I A K A G I T A T L N A R
P24279	*Sc*Mcm3	490	M E Q Q T V T I A K A G I H T T L N A R
P25205	*Hs*Mcm3	426	M E Q G R V T I A K A G I H A R L N A R
P30666	*Sp*Mcm3	437	M E Q Q T V T I A K A G I H T S L N A R
Q9XYU1	*Dm*Mcm3	421	M E Q G R V T I S K A G I H A S L N A R
Q9XVR7	*Ce*Mcm3	429	M E Q G R V T I S K A G I H A K L N A R
P49739	*Xl*Mcm3	426	M E Q G R V T I A K A G I Q A R L N A R
Q5RIC5	*Dr*Mcm3	425	M E Q G R V T I A K A G I H A R L N A R
P25206	*Mm*Mcm3	426	M E Q G R V T I A K A G I H A R L N A R
Q9FL33	*At*Mcm3	416	M E Q Q T V T I A K A G I H A S L N A R
Q54VI9	*Dd*Mcm3	440	M E Q Q T V T I S K A G I H A S L N A R
Q24849	*Eh*Mcm3	310	M E Q Q T V T V Q K A G I H T A L N A R
EES99567.1	*Gd*Mcm3	476	L E Q Q S I S I S K A G L H C T L N A R

Supplementary Fig. 19: Sequence alignment of the MCM pre-sensor-1-β hairpin.

The alignment has archaeal sequences followed by eukaryotic sequences grouped by subunit type and placed in sequential order about the ring as in Fig. 7d. Residues with 95% conservation are in bold with cyan shading. A conserved positive residue that interacts with a DNA phosphate group is in bold and shaded. This residue is lysine in all instances (shaded magenta) except *Dm*Mcm6 where it is arginine (shaded light magenta). For *Tk*MCM, residues 554-888 have been removed to account for the intein removed from the mature protein. Archaeal MCM: *Sso=Saccharolobus solfataricus* ; *Pf=Pyrococcus furiosus*; *Sa=Sulfolobus acidocaldarius*; *Mt=Methanothermobacter thermautotrophicus*; *Ap=Aeropyrum pernix*; *Tk=Thermococcus kodakarensis*. Eukaryotic Mcm2-7: *Sc=Saccharomyces cerevisiae*; *Hs=Homo sapiens*; *Sp=Schizosaccharomyces pombe*; *Dm=Drosophila melanogaster*; *Ce=Caenorhabditis elegans*; *Xl=Xenopus laevis*; *Dr=Danio rerio*; *Mm=Mus musculus*; *At=Arabidopsis thaliana*; *Dd=Dictyostelium discoideum*; *Eh=Entamoeba histolytica* ATCC 30459; *Gd=Giardia duodenalis* ATCC 50581. Accession numbers are provided to the left of each sequence.

BPV1	P03116	496	LDGYPVSIDRKHKAAVQI	HPV21	P50759	494	LDGHVVSLDCKHKAPMQT
HPV RTRX7	O40619	495	LDGHFVSLDCKYKAPVQT	HPV22	P50760	499	LDGNMVSLDMKHRAPCQM
HPV1a	P03111	503	LDGNTICIDLKHRAPQQI	HPV23	P50761	498	LDGNVVSLDMKHRAPCQM
CRPV	P03112	494	LDGNPISVDLKHKAPIEI	HPV24	P50762	498	LDGHLVSLDCKHKAPIQI
HPV6b	P03113	541	LDGNPMSIDRKHKALTLI	HPV28	P50763	553	LDGNQVCIDRKHRALLQL
HPV16	P03114	540	LDGNLVSMDVKHRPLVQL	HPV29	P50764	551	LDGNVMCIDRKHRSLLQL
DPV	P03117	503	LDGYPFGIDRKHNTAVQM	HPV70	P50765	542	LDGNPISLDRKHRHLIQI
HPV11	P04014	541	LDGNPMSIDRKHRALTLI	HPV36	P50808	495	LDGHYVSLDCKYKAPMQT
HPV8	P06420	494	LDGHVVSLDCKYKAPMQI	PCPV-1C	P81174	540	LDGNPMSIDRKHKSLALI
HPV33	P06421	533	LDGNEISIDVKHRALVQL	BPV3	Q8BDD7	505	LDGTPVSLDMKHRAPMQI
APV	P06455	112	LDGNAICIDCKHRAPVQT	HPV69	Q9JH49	526	LDGNPCCIDRKHRSLIQV
HPV18	P06789	547	LDGNPISIDRKHKPLIQL	BPV6	Q705F4	501	LDGTPVSLDMKHRAPIQI
HPV5	P06920	497	LDGHYVSLDCKYRAPTQM	BPV5	Q705G8	515	FDGYSVCIDRKHKNAVQI
BPV4	P08344	500	LDGTPVSLDMKHRAPLQI	HPV19	Q02048	495	LDGHVVSLDCKHKAPIQT
BPV2	P11298	495	LDGYPVSIDRKHKAAVQI	HPV25	Q02049	495	LDGHYVSLDCKHKAPMQT
EEPV	P11328	501	LDGYPVCIDRKHKSAVQL	HPV13	Q02261	538	LDGNPMSIDRKHKSLALI
HPV31	P17382	520	LDGNPVSIDVKHKALMQL	PCPV-1	Q02262	540	LDGNPMSIDRKHKSLALI
HPV57	P22153	534	LDGNPFSIDRKHKTLLQI	HPV17	Q02512	500	LDGNVVCLDLKHRAPCQI
RhPV1	P22154	516	LDGNPISVDRKHKNLVQM	HPV9	Q05111	496	LDGNYVCLDMKHRAPCQM
HPV47	P22419	496	LDGHFVSLDCKYRAPMQT	HPV30	Q05112	522	LDGNPVSLDRKHKQLVQI
HPV39	P24829	537	LDGYAISLDRKYKSLLQM	HPV7	Q05133	539	LDGNPTSIDRKHKSLAVI
HPV2a	P25481	534	LDGHPVSIDRKHKTLLQL	HPV12	Q05134	495	LDGHFVSLDCKYKAPVQI
HPV5b	P26542	497	LDGHYVSLDCKYRAPTQM	HPV15	Q05135	493	LDGGVVCLDMKHRAPCQI
HPV58	P26543	533	LDGNDISIDVKHRALVQL	HPV4	Q07846	491	LDGNPMCIDAKHRAPQQL
HPV51	P26544	526	LDGNPCSIDRKHRSLIQL	HPV63	Q07847	509	LDGNPICVDLKHKAPQQI
HPV35	P27220	526	LDGNPISLDVKHKALVQL	HPV65	Q07848	490	LDGNPMCIDAKHRAPQQL
HPV42	P27221	534	LDGNPCSIDRKHKALTVV	HPV37	Q80902	500	LDGNFVCLDLKHRAPCQI
HPV41	P27551	506	LDGYEISIDAKHRNPMQT	HPV38	Q80909	495	LDGNLVSLDIKHKAPCQM
HPV3	P36719	550	LDGNQVCIDRKHRALLQL	HPV44	Q80915	535	LDGNPMTIDRKHKSLALI
HPV10	P36720	572	LDGNQICVDRKHRALLQL	HPV48	Q80922	485	FDGNTFCLDIKHKNLQQT
HPV14	P36721	496	LDGHYVSLDCKHKAPIQT	HPV60	Q80943	500	LDGNHISLDLKHKAPLQI
HPV26	P36722	530	LDGNPCCIDRKHRSLLQV	HPV61	Q80950	539	LDGNAISIDRKHRNLTQL
HPV27	P36723	534	LDGNPVSIDRKHKTLLQL	HPV66	Q80957	521	LDGNPISLDRKHKQLVQI
HPV32	P36724	533	LDGNPCSIDRKHKALTVV	HPV54	Q81020	523	LDGNPMCFDRKHRAMVQT
HPV34	P36725	538	LDGNPMCLDRKHKHLLQI	HPV59	Q81966	534	LDGNPISVDRKHRHLVQI
HPV40	P36727	540	LDGNPTSIDRKHKPLAVI	HPV72	Q81999	539	LDGNAISIDRKHRNLTQL
HPV45	P36728	533	LDGNPISIDRKHKPLLQL	HPV73	Q82007	541	LDGNPICLDRKHKNLLQV
HPV49	P36729	500	LDGNVVSVDCKHKTPMQI	HPV6a	Q84293	541	LDGNPMSIDRKHKALTLI
HPV52	P36730	536	LDGNDISVDVKHRALVQI	MnPV	Q84356	492	MDGNPLSIDCKYRTPVQV
HPV20	P50758	496	LDGHYVSLDCKHKAPIQT	COPV	Q89536	489	LDGNTFSVDCKHKAPLQL

Supplementary Fig. 20: Sequence alignment of the Papillomavirus E1 pre-sensor-1-β hairpin.

All 80 Prosite[137] reviewed sequences are shown in two adjacent groups of 40. Accession numbers and strain abbreviations are provided. Residues with 95% conservation are in bold with cyan shading. A conserved lysine that interacts with a DNA phosphate group is in bold and shaded magenta. A conserved aromatic group that stacks on a DNA base and sugar directly follows the conserved lysine. This residue is histidine in 71 cases (in bold and shaded yellow) and tyrosine in 9 cases (in bold and shaded orange).

Supplementary Table 1: Cryo-EM statistics for merged particle Class 1 structures.

	Merged MCM:DNA Class 1a (EMD-49806) (PDB 9NUH)	Merged MCM:DNA Class 1b (EMD-49807) (PDB 9NUI)	Merged MCM:DNA Class 1c (EMD-49808) (PDB 9NUJ)
**Data collection and processing**			
Magnification	81,000	81,000	81,000
Voltage (kV)	300	300	300
Electron exposure (e–/Å^2)	76.5 / 78.22	76.5 / 78.22	76.5 / 78.22
Defocus range (μm)	-0.8 to -1.8	-0.8 to -1.8	-0.8 to -1.8
Pixel size (Å)	1.08	1.08	1.08
Symmetry imposed	C1	C1	C1
Initial particle images (no.)	30,000,296	30,000,296	30,000,296
Final particle images (no.)	101,708	60,650	30,802
Map resolution (Å)	2.92	3.24	3.44
FSC threshold	0.143	0.143	0.143
Map resolution range (Å)	2.92	3.24	3.44
**Refinement**			
Initial model used (PDB code)	4R7Y	4R7Y	4R7Y
Model resolution (Å)	2.92	3.24	3.44
FSC threshold			
Model resolution range (Å)	2.92	3.24	3.44
Map sharpening *B* factor (Å^2)	-98.0	unsharpened	unsharpened
Model composition			
Non-hydrogen atoms	29431	27219	27096
Protein residues	3546	3214	3214
DNA residues	46	68	62
Ligands	18	14	14
B factors (Å^2)			
Protein	84.759	215.151	186.991
DNA	178.934	350.052	316.968
Ligand	84.031	199.973	178.266
R.m.s. deviations			
Bond lengths (Å)	0.0035	0.0024	0.0036
Bond angles (°)	0.58	0.54	0.56
Validation			
MolProbity score	1.86	1.91	2.06
Clashscore	3.65	3.90	5.65
Poor rotamers (%)	2.58	2.72	2.48
Ramachandran plot			
Favored (%)	94.15	93.92	92.92
Allowed (%)	5.85	6.05	6.99
Disallowed (%)	0	0.03	0.09

Supplementary Table 2: Cryo-EM statistics for merged particle Class 2 and Class 3 structures.

	Merged MCM:DNA Class 2a (EMD-49809) (PDB 9NUK)	Merged MCM:DNA Class 2b (EMD-49810) (PDB 9NUL)	Merged MCM:DNA Class 3 (EMD-49811) (PDB 9NUM)
**Data collection and processing**			
Magnification	81,000	81,000	81,000
Voltage (kV)	300	300	300
Electron exposure (e–/Å^2)	76.5 / 78.22	76.5 / 78.22	76.5 / 78.22
Defocus range (μm)	-0.8 to -1.8	-0.8 to -1.8	-0.8 to -1.8
Pixel size (Å)	1.08	1.08	1.08
Symmetry imposed	C1	C1	C1
Initial particle images (no.)	30,000,296	30,000,296	30,000,296
Final particle images (no.)	384,077	471,221	50,839
Map resolution (Å)	2.70	2.50	3.67
FSC threshold	0.143	0.143	0.143
Map resolution range (Å)	2.70	2.50	3.67
**Refinement**			
Initial model used (PDB code)	4R7Y	4R7Y	4R7Y
Model resolution (Å)	2.70	2.50	3.67
FSC threshold			
Model resolution range (Å)	2.70	2.50	3.67
Map sharpening *B* factor (Å^2)	unsharpened	unsharpened	unsharpened
Model composition			
Non-hydrogen atoms	29359	29478	34589
Protein residues	3576	3576	4172
DNA residues	30	36	52
Ligands	18	18	21
B factors (Å^2)			
Protein	163.938	142.609	216.528
DNA	297.740	262.884	315.035
Ligand	145.047	115.225	180.296
R.m.s. deviations			
Bond lengths (Å)	0.0050	0.0022	0.0031
Bond angles (°)	0.58	0.52	0.59
Validation			
MolProbity score	1.61	1.35	2.07
Clashscore	3.56	2.62	6.59
Poor rotamers (%)	1.28	0.57	2.38
Ramachandran plot			
Favored (%)	94.43	95.61	93.63
Allowed (%)	5.49	4.28	6.37
Disallowed (%)	0.08	0.11	0.00

Supplementary Table 3: Cryo-EM statistics for Class 1 structures with DNA1.

	MCM:DNA1 Class 1a (EMD-49812) (PDB 9NUN)	MCM:DNA1 Class 1b (EMD-49813) (PDB 9NUO)	MCM:DNA1 Class 1c (EMD-49814) (PDB 9NUP)
**Data collection and processing**			
Magnification	81,000	81,000	81,000
Voltage (kV)	300	300	300
Electron exposure (e–/Å^2)	76.5	76.5	76.5
Defocus range (μm)	-0.8 to -1.8	-0.8 to -1.8	-0.8 to -1.8
Pixel size (Å)	1.08	1.08	1.08
Symmetry imposed	C1	C1	C1
Initial particle images (no.)	15,146,308	15,146,308	15,146,308
Final particle images (no.)	40,953	32,139	15,877
Map resolution (Å)	3.36	3.58	3.90
FSC threshold	0.143	0.143	0.143
Map resolution range (Å)	3.36	3.58	3.90
**Refinement**			
Initial model used (PDB code)	4R7Y	4R7Y	4R7Y
Model resolution (Å)	3.36	3.58	3.90
FSC threshold			
Model resolution range (Å)	3.36	3.58	3.90
Map sharpening *B* factor (Å^2)	unsharpened	unsharpened	unsharpened
Model composition			
Non-hydrogen atoms	29677	27301	27137
Protein residues	3546	3214	3214
DNA residues	58	72	64
Ligands	18	14	14
B factors (Å^2)			
Protein	184.500	180.054	211.926
DNA	276.860	364.485	323.286
Ligand	188.774	164.886	197.044
R.m.s. deviations			
Bond lengths (Å)	0.0023	0.0029	0.0038
Bond angles (°)	0.54	0.54	0.58
Validation			
MolProbity score	1.52	1.89	2.26
Clashscore	2.98	4.46	7.77
Poor rotamers (%)	1.32	2.05	3.11
Ramachandran plot			
Favored (%)	95.09	93.33	92.64
Allowed (%)	4.91	6.64	7.33
Disallowed (%)	0.00	0.03	0.03

Supplementary Table 4: Cryo-EM statistics for Class 2 and Class 3 structures with DNA1.

	MCM:DNA1 Class 2a (EMD-49815) (PDB 9NUQ)	MCM:DNA1 Class 2b (EMD-49816) (PDB 9NUR)	MCM:DNA1 Class 3 (EMD-49817) (PDB 9NUS)
**Data collection and processing**			
Magnification	81,000	81,000	81,000
Voltage (kV)	300	300	300
Electron exposure (e–/Å^2)	76.5	76.5	76.5
Defocus range (μm)	-0.8 to -1.8	-0.8 to -1.8	-0.8 to -1.8
Pixel size (Å)	1.08	1.08	1.08
Symmetry imposed	C1	C1	C1
Initial particle images (no.)	15,146,308	15,146,308	15,146,308
Final particle images (no.)	148,429	196,176	25,549
Map resolution (Å)	3.13	2.99	4.04
FSC threshold	0.143	0.143	0.143
Map resolution range (Å)	3.13	2.99	4.04
**Refinement**			
Initial model used (PDB code)	4R7Y	4R7Y	4R7Y
Model resolution (Å)	3.13	2.99	4.04
FSC threshold			
Model resolution range (Å)	3.13	2.99	4.04
Map sharpening *B* factor (Å^2)	-135.4	-134.6	unsharpened
Model composition			
Non-hydrogen atoms	29605	29724	34589
Protein residues	3576	3576	4172
DNA residues	42	48	52
Ligands	18	18	21
B factors (Å^2)			
Protein	71.904	71.131	229.271
DNA	170.712	174.042	284.636
Ligand	46.435	71.131	203.091
R.m.s. deviations			
Bond lengths (Å)	0.0025	0.0023	0.0040
Bond angles (°)	0.54	0.54	0.61
Validation			
MolProbity score	1.52	1.32	1.95
Clashscore	2.97	2.01	9.56
Poor rotamers (%)	1.34	1.18	0.00
Ramachandran plot			
Favored (%)	95.19	95.72	93.15
Allowed (%)	4.73	4.17	6.85
Disallowed (%)	0.08	0.11	0.00

Supplementary Table 5: Cryo-EM statistics for Class 1 structures with DNA2.

	MCM:DNA2 Class 1a (EMD-49818) (PDB 9NUT)	MCM:DNA2 Class 1b (EMD-49819) (PDB 9NUU)	MCM:DNA2 Class 1c (EMD-49820) (PDB 9NUV)
**Data collection and processing**			
Magnification	81,000	81,000	81,000
Voltage (kV)	300	300	300
Electron exposure (e–/Å^2)	78.22	78.22	78.22
Defocus range (μm)	-0.8 to -1.8	-0.8 to -1.8	-0.8 to -1.8
Pixel size (Å)	1.08	1.08	1.08
Symmetry imposed	C1	C1	C1
Initial particle images (no.)	14,853,988	14,853,988	14,853,988
Final particle images (no.)	60,755	28,511	14,925
Map resolution (Å)	2.93	3.38	3.51
FSC threshold	0.143	0.143	0.143
Map resolution range (Å)	2.93	3.38	3.51
**Refinement**			
Initial model used (PDB code)	4R7Y	4R7Y	4R7Y
Model resolution (Å)	2.93	3.38	3.51
FSC threshold			
Model resolution range (Å)	2.93	3.38	3.51
Map sharpening *B* factor (Å^2)	unsharpened	unsharpened	unsharpened
Model composition			
Non-hydrogen atoms	29513	27178	26891
Protein residues	3546	3214	3214
DNA residues	50	66	52
Ligands	18	14	14
B factors (Å^2)			
Protein	161.887	201.156	209.163
DNA	237.249	318.022	278.559
Ligand	162.576	185.429	196.905
R.m.s. deviations			
Bond lengths (Å)	0.0039	0.0034	0.0034
Bond angles (°)	0.61	0.56	0.57
Validation			
MolProbity score	1.62	2.06	2.11
Clashscore	4.41	5.45	6.71
Poor rotamers (%)	1.03	2.55	2.41
Ramachandran plot			
Favored (%)	94.26	92.79	93.05
Allowed (%)	5.74	7.17	6.92
Disallowed (%)	0.00	0.03	0.03

Supplementary Table 6: Cryo-EM statistics for Class 2 and Class 3 structures with DNA2.

	MCM:DNA2 Class 2a (EMD-49821) (PDB 9NUW)	MCM:DNA2 Class 2b (EMD-49822) (PDB 9NUX)	MCM:DNA2 Class 3 (EMD-49823) (PDB 9NUY)
**Data collection and processing**			
Magnification	81,000	81,000	81,000
Voltage (kV)	300	300	300
Electron exposure (e–/Å^2)	78.22	78.22	78.22
Defocus range (μm)	-0.8 to -1.8	-0.8 to -1.8	-0.8 to -1.8
Pixel size (Å)	1.08	1.08	1.08
Symmetry imposed	C1	C1	C1
Initial particle images (no.)	14,853,988	14,853,988	14,853,988
Final particle images (no.)	235,648	275,045	25,290
Map resolution (Å)	2.65	2.45	3.52
FSC threshold	0.143	0.143	0.143
Map resolution range (Å)	2.65	2.45	3.52
**Refinement**			
Initial model used (PDB code)	4R7Y	4R7Y	4R7Y
Model resolution (Å)	2.65	2.45	3.52
FSC threshold			
Model resolution range (Å)	2.65	2.45	3.52
Map sharpening *B* factor (Å^2)	-102.2	-91.6	unsharpened
Model composition			
Non-hydrogen atoms	29194	29312	34589
Protein residues	3576	3576	4172
DNA residues	22	28	52
Ligands	18	18	21
B factors (Å^2)			
Protein	67.087	58.720	217.285
DNA	148.645	126.134	325.098
Ligand	50.241	31.696	177.903
R.m.s. deviations			
Bond lengths (Å)	0.0040	0.0046	0.0027
Bond angles (°)	0.59	0.65	0.61
Validation			
MolProbity score	1.71	1.68	2.05
Clashscore	3.22	2.94	6.69
Poor rotamers (%)	1.98	1.95	2.30
Ramachandran plot			
Favored (%)	94.62	94.54	93.85
Allowed (%)	5.29	5.35	6.15
Disallowed (%)	0.08	0.11	0.00

Supplementary Table 7: Top 30 Mcm2-7 models nearest to Class 1a based on RMSD of the six ATPase core structures.

PDB ID	Resolution	Setting	RMSD	CMG?
8S0F	4.1	SETTING 3	3.481	No
8S0F	4.1	SETTING 6	3.487	No
8S0F	4.1	SETTING 2	3.494	No
8S0F	4.1	SETTING 1	3.504	No
8S0F	4.1	SETTING 4	3.506	No
8S0F	4.1	SETTING 5	3.511	No
8KG9	4.52	SETTING 3	3.715	Yes
8KG9	4.52	SETTING 4	3.722	Yes
8KG9	4.52	SETTING 2	3.726	Yes
8KG9	4.52	SETTING 1	3.733	Yes
8KG9	4.52	SETTING 6	3.738	Yes
8KG9	4.52	SETTING 5	3.739	Yes
6XTY	6.77	SETTING 6	3.871	Yes
6XTY	6.77	SETTING 3	3.878	Yes
6XTY	6.77	SETTING 5	3.885	Yes
6XTY	6.77	SETTING 1	3.888	Yes
6XTY	6.77	SETTING 4	3.888	Yes
6XTY	6.77	SETTING 2	3.893	Yes
6RAZ	4.46	SETTING 5	3.918	Yes
6RAZ	4.46	SETTING 6	3.926	Yes
6RAZ	4.46	SETTING 4	3.936	Yes
6RAZ	4.46	SETTING 3	3.937	Yes
6RAZ	4.46	SETTING 1	3.941	Yes
8S0E	3.8	SETTING 6	3.946	No
6RAZ	4.46	SETTING 2	3.95	Yes
8S0E	3.8	SETTING 2	3.951	No
8S0E	3.8	SETTING 3	3.953	No
8S0E	3.8	SETTING 1	3.965	No
8S0E	3.8	SETTING 5	3.967	No
8S0E	3.8	SETTING 4	3.974	No

Supplementary Table 8: Top 30 Mcm2-7 models nearest to Class 2b based on RMSD of the six ATPase core structures.

PDB ID	Resolution	Setting	RMSD	CMG?	
9E2W	3.3	SETTING 1	1.288	Yes	
9E2Y	3.2	SETTING 1	1.308	Yes	
8KG9	4.52	SETTING 2	1.331	Yes	
8KG8	4.23	SETTING 1	1.457	Yes	
8XGC	3.7	SETTING 1	1.483	Yes	
8KG6	3.07	SETTING 1	1.487	Yes	
6PTN	5.8	SETTING 1	1.495	Yes	
6PTO	7	SETTING 1	1.495	Yes	
**9E2X**	**3.5**	**SETTING 5**	**1.5**	**Yes**	**See Fig. 8**
9BCX	6.1	SETTING 6	1.527	No	
6U0M	3.9	SETTING 1	1.528	Yes	
8P63	3.7	SETTING 1	1.54	Yes	
8P62	3.9	SETTING 1	1.565	Yes	
8P5E	3.9	SETTING 1	1.573	Yes	
5U8S	6.1	SETTING 1	1.608	Yes	
**7Z13**	**3.4**	**SETTING 1**	**1.641**	**Yes**	**See Fig. 6**
7QHS	3.3	SETTING 1	1.643	Yes	
**9GJW**	**3.3**	**SETTING 5**	**1.649**	**No**	**See Fig. 8**
6SKL	3.7	SETTING 1	1.663	Yes	
6RAX	3.99	SETTING 1	1.681	Yes	
8W7M	4.12	SETTING 4	1.692	Yes	
**7PMK**	**3.2**	**SETTING 1**	**1.708**	**Yes**	**See Fig. 6**
6XTY	6.77	SETTING 4	1.709	Yes	
6HV9	4.98	SETTING 1	1.743	Yes	
5U8T	4.9	SETTING 1	1.789	Yes	
9E2Z	2.6	SETTING 1	1.854	Yes	
6RAZ	4.46	SETTING 4	1.932	Yes	
**7PMN**	**3.2**	**SETTING 6**	**1.96**	**Yes**	**See Fig. 6**
6RAY	4.28	SETTING 5	1.962	Yes	
8KG9	4.52	SETTING 1	1.967	Yes	

28 of Top 30 are CMG structures, and these are heavily overweighted in adopting Setting 1:

Setting 1: 21 (75%)

Setting 2: 1 (3.6 %)

Setting 3: 0 (0 %)

Setting 4: 3 (10.7 %)

Setting 5: 2 (7.1 %)

Setting 6: 1 (3.6 %)

Models selected for comparison in the manuscript are highlighted in bold.
