# Supplementary material for: Archaeal and eukaryotic MCM rings sequentially melt DNA for replication initiation
Source: Nat Commun. 2026 Mar 31;17:4681. doi: 10.1038/s41467-026-70961-8 (PMC13201583; doi:10.1038/s41467-026-70961-8)
Supplement: Supplementary file 2 — Description of Additional Supplementary Files [file 41467_2026_70961_MOESM2_ESM.pdf]

Description of Additional Supplementary Files

File name: Supplementary Movie 1
Description: Comparison of eclipsed and staggered conformations. The eclipsed conformation of Class 1a was morphed to the staggered conformation of Class 2b in the top middle panel. Several comparative transformations were generated for eukaryotic MCM structures. All panels are viewed down the central channel from the N-tier side with an N-tier frame of reference that minimizes N-tier motion. All transformations behave qualitatively similarly with the ATPase C-tier rotating counter-clockwise. All morphs were generated with the rigimol routine of PyMOL[86] and are intended to help convey how the eclipsed and staggered conformations differ and not to imply specific molecular dynamics or that the specific structural forms would directly transform. Depicted eclipsed structures: Class 1a, PDB 5XF8[93], PDB 6WGG[91], PDB 5V8F[92], PDB 9GJW[89], PDB 9BCX[90]. Depicted staggered structures: Class 2b, PDB 7Z13[71].

File name: Supplementary Movie 2
Description: Model for transformation of the Mcm2-7 double hexamer encircling dsDNA to two CMGs encircling melted DNA. The initial double-hexamer has two MCM rings in a stable Staggered Form I configuration. The double-hexamer interface breaks with Hexamer 2 (bottom) sliding away from hexamer 1. The implicit loss of Hexamer 2:DNA interactions allows the internal DNA bubble of PDB 7W1Y[99] to re-anneal. Next, each hexamer follows the sequential melting sequence of **Fig. 9a** with each hexamer likely undergoing the transformation independently. The first melting step is based on PDB 9GJW[89] rather than PDB 9E2X[98] because 9GJW[89] is the closest ATPase tier structural match that includes encircled dsDNA (see **Fig. 8**). The models and morphs are to provide conceptual visualization and not precise molecular dynamics. The protein model of PDB 7W1Y[99] was converted to a yeast model to enable direct residue morphing. Molecular morphs were generated with the rigimol routine of PyMOL[86]. Structures used: PDB 7W1Y[99], PDB 9GJW[89], PDB 7PMN[96], PDB 7Z13[71]. Cdc45 and GINS are not part of the morph because they are not common to the starting and ending states.

File name: Supplementary Movie 3
Description: Proposed mechanism for extension of DNA melting based on an external pulling force. DNA melting can be extended if an external force pulls the DNA with respect to the stable Staggered Form II structure with a fixed DNA-melting wedge. For each discrete step, the DNA slides one increment along the DNA-binding hairpins analogous to a gear slip (see **Fig. 9**). Molecular images for the right panel were generated in PyMOL[86]. A set of nucleotides are highlighted in green to highlight the transformation from base-paired to melted and DNA movement with respect to the ring. In the left cartoon, unpaired bases are outlined in red.

File name: Supplementary Movie 4
Description: Proposed mechanism for an external wedge to provide an external force to extend DNA melting. The external force to pull the DNA relative to a static MCM ring could be provided by an external wedge, perhaps Mcm10, that pries the two hexamers apart. This wedge may also bind and stabilize exposed ssDNA and assist to remodel the MCM N-tier to enable subsequent exit of the strand to be excluded from the MCM ring during active replication (see **Fig. 9**). Both hexamers persist in a stable Staggered Form II structure with a fixed DNA-melting aromatic wedge. In this depiction, Hexamer 1 (top) and its associated DNA remain constant, and the wedge pushes hexamer 2 (bottom) away from Hexamer 1. This action melts DNA in its wake as illustrated in Supplementary Movie 3 (see also **Fig. 9**). A set of nucleotides are in green to highlight the transformation from base-paired to melted and the movement of DNA with respect to the ring. Coordinates for the MCM complex and much of the DNA are derived PDB 7Z13[71]. Cdc45 and GINS are excluded to focus on MCM:DNA. Molecular morphs were generated with the rigimol routine of PyMOL[86].
