# Supplementary material for: Archaeal and eukaryotic MCM rings sequentially melt DNA for replication initiation
Source: Nat Commun. 2026 Mar 31;17:4681. doi: 10.1038/s41467-026-70961-8 (PMC13201583; doi:10.1038/s41467-026-70961-8)
Supplement: Supplementary file 7 — Reporting Summary [file 41467_2026_70961_MOESM7_ESM.pdf]

nature portfolio

Corresponding author(s): Eric J. Enemark

Last updated by author(s): Feb 12, 2026

Reporting Summary

Nature Portfolio wishes to improve the reproducibility of the work that we publish. This form provides structure for consistency and transparency in reporting. For further information on Nature Portfolio policies, see our Editorial Policies and the Editorial Policy Checklist.

Statistics

For all statistical analyses, confirm that the following items are present in the figure legend, table legend, main text, or Methods section.

n/a	Confirmed	
☐	☒	The exact sample size (*n*) for each experimental group/condition, given as a discrete number and unit of measurement
☒	☐	A statement on whether measurements were taken from distinct samples or whether the same sample was measured repeatedly
☒	☐	The statistical test(s) used AND whether they are one- or two-sided<br>*Only common tests should be described solely by name; describe more complex techniques in the Methods section.*
☒	☐	A description of all covariates tested
☒	☐	A description of any assumptions or corrections, such as tests of normality and adjustment for multiple comparisons
☒	☐	A full description of the statistical parameters including central tendency (e.g. means) or other basic estimates (e.g. regression coefficient) AND variation (e.g. standard deviation) or associated estimates of uncertainty (e.g. confidence intervals)
☒	☐	For null hypothesis testing, the test statistic (e.g. *F*, *t*, *r*) with confidence intervals, effect sizes, degrees of freedom and *P* value noted<br>*Give P values as exact values whenever suitable.*
☒	☐	For Bayesian analysis, information on the choice of priors and Markov chain Monte Carlo settings
☒	☐	For hierarchical and complex designs, identification of the appropriate level for tests and full reporting of outcomes
☒	☐	Estimates of effect sizes (e.g. Cohen's *d*, Pearson's *r*), indicating how they were calculated

Our web collection on statistics for biologists contains articles on many of the points above.

Software and code

Policy information about availability of computer code

Data collection	SerialEM, MotionCor2
Data analysis	Cryo-EM analysis: Cryosparc (v2.11.0, v2.14.2, v3.3.1, v4.4.1, v4.5.3), Relion (3.0-beta-2, 3.07), cisTEM 1.0.0-beta, CTFind, PyEM 0.5, Coot, Phenix 1.21.2_5419, Chimera 1.18 build 42531, Chimera 1.19 build 42556, ChimeraX 1.8, PyMOL (2.5.8, 3.1.6.1).<br>Dihedral comparison: PyMOL executed with elementary bash scripting. Scripts, description, and results are provided at: [https://doi.org/10.5281/zenodo.18487880] (MCM Inter-Tier Dihedral Calculations)<br>Quantitative RMSD analysis: PyMOL executed with elementary bash scripting. Scripts, description, and results are provided at: [https://doi.org/10.5281/zenodo.18487583] (MCM Setting RMSD Calculations)

For manuscripts utilizing custom algorithms or software that are central to the research but not yet described in published literature, software must be made available to editors and reviewers. We strongly encourage code deposition in a community repository (e.g. GitHub). See the Nature Portfolio guidelines for submitting code & software for further information.

Data

Policy information about availability of data

All manuscripts must include a data availability statement. This statement should provide the following information, where applicable:

- Accession codes, unique identifiers, or web links for publicly available datasets
- A description of any restrictions on data availability
- For clinical datasets or third party data, please ensure that the statement adheres to our policy

The molecular coordinates and maps have been deposited at the PDB and EMDB with the following accession codes:
9NUH / EMD-49806 SsoPfMCM:DNA class 1a from merged particles
9NUI / EMD-49807 SsoPfMCM:DNA class 1b from merged particles
9NUJ / EMD-49808 SsoPfMCM:DNA class 1c from merged particles
9NUK / EMD-49809 SsoPfMCM:DNA class 2a from merged particles
9NUL / EMD-49810 SsoPfMCM:DNA class 2b from merged particles
9NUM / EMD-49811 SsoPfMCM:DNA class 3 from merged particles
9NUN / EMD-49812 SsoPfMCM:DNA class 1a from DNA 1
9NUO / EMD-49813 SsoPfMCM:DNA class 1b from DNA 1
9NUP / EMD-49814 SsoPfMCM:DNA class 1c from DNA 1
9NUQ / EMD-49815 SsoPfMCM:DNA class 2a from DNA 1
9NUR / EMD-49816 SsoPfMCM:DNA class 2b from DNA 1
9NUS / EMD-49817 SsoPfMCM:DNA class 3 from DNA 1
9NUT / EMD-49818 SsoPfMCM:DNA class 1a from DNA 2
9NUU / EMD-49819 SsoPfMCM:DNA class 1b from DNA 2
9NUV / EMD-49820 SsoPfMCM:DNA class 1c from DNA 2
9NUW / EMD-49821 SsoPfMCM:DNA class 2a from DNA 2
9NUX / EMD-49822 SsoPfMCM:DNA class 2b from DNA 2
9NUY / EMD-49823 SsoPfMCM:DNA class 3 from DNA 2

Research involving human participants, their data, or biological material

Policy information about studies with human participants or human data. See also policy information about sex, gender (identity/presentation), and sexual orientation and race, ethnicity and racism.

Reporting on sex and gender	N/A
Reporting on race, ethnicity, or other socially relevant groupings	N/A
Population characteristics	N/A
Recruitment	N/A
Ethics oversight	N/A

Note that full information on the approval of the study protocol must also be provided in the manuscript.

Field-specific reporting

Please select the one below that is the best fit for your research. If you are not sure, read the appropriate sections before making your selection.

☒ Life sciences ☐ Behavioural & social sciences ☐ Ecological, evolutionary & environmental sciences

For a reference copy of the document with all sections, see nature.com/documents/nr-reporting-summary-flat.pdf

Life sciences study design

All studies must disclose on these points even when the disclosure is negative.

Sample size	Number of micrographs were determined by the number that could be collected within defined microscope time.
Data exclusions	Cryo-EM data processing began with all images included. See Supplementary Fig. 1-7 for workflow details.
Replication	To verify that the MCM:DNA structures are general and not associated with a specific DNA sequence or DNA reagent, structures were determined for samples generated from two independent DNA sequences. The underlying structural classes of these two samples are highly similar, which enabled particle merging to generate higher resolution versions of each structural class.

Randomization: As standard, all micrographs are imported for analysis, and thus randomization is not relevant.

Blinding: As standard, all micrographs are imported for analysis, and thus blinding is not relevant.

Reporting for specific materials, systems and methods

We require information from authors about some types of materials, experimental systems and methods used in many studies. Here, indicate whether each material, system or method listed is relevant to your study. If you are not sure if a list item applies to your research, read the appropriate section before selecting a response.

Materials & experimental systems

n/a	Involved in the study
☒	☐ Antibodies
☒	☐ Eukaryotic cell lines
☒	☐ Palaeontology and archaeology
☒	☐ Animals and other organisms
☒	☐ Clinical data
☒	☐ Dual use research of concern
☒	☐ Plants

Methods

n/a	Involved in the study
☒	☐ ChIP-seq
☒	☐ Flow cytometry
☒	☐ MRI-based neuroimaging

Plants

Seed stocks: N/A

Novel plant genotypes: N/A

Authentication: N/A
